# A functional genomics pipeline identifies pleiotropy and cross-tissue effects within obesity-associated GWAS loci

**DOI:** 10.1038/s41467-021-25614-3

**Published:** 2021-09-06

**Authors:** Amelia C. Joslin, Débora R. Sobreira, Grace T. Hansen, Noboru J. Sakabe, Ivy Aneas, Lindsey E. Montefiori, Kathryn M. Farris, Jing Gu, Donna M. Lehman, Carole Ober, Xin He, Marcelo A. Nóbrega

**Affiliations:** 1 Department of Human Genetics, University of Chicago, Chicago, IL USA; 2 Department of Medicine, University of Texas Health Science Center at San Antonio, San Antonio, TX USA

**Keywords:** Functional genomics, Gene regulation, Epigenomics, Obesity

## Abstract

Genome-wide association studies (GWAS) have identified many disease-associated variants, yet mechanisms underlying these associations remain unclear. To understand obesity-associated variants, we generate gene regulatory annotations in adipocytes and hypothalamic neurons across cellular differentiation stages. We then test variants in 97 obesity-associated loci using a massively parallel reporter assay and identify putatively causal variants that display cell type specific or cross-tissue enhancer-modulating properties. Integrating these variants with gene regulatory information suggests genes that underlie obesity GWAS associations. We also investigate a complex genomic interval on 16p11.2 where two independent loci exhibit megabase-range, cross-locus chromatin interactions. We demonstrate that variants within these two loci regulate a shared gene set. Together, our data support a model where GWAS loci contain variants that alter enhancer activity across tissues, potentially with temporally restricted effects, to impact the expression of multiple genes. This complex model has broad implications for ongoing efforts to understand GWAS.

## Abstract (web summary)

Many genetic loci have been linked to obesity, but knowledge of their functional mechanisms is limited. Here, the authors perform reporter assays and temporal functional genomics data generation to characterize obesity genetic loci and find that loci often harbor multiple functional variants.

## Introduction

While genome-wide association studies (GWAS) have been instrumental in associating genetic variation to disease, functional studies delineating specific causal variants or effector genes of these associations have yet to become commonplace. Current evidence indicates that a significant number of associated variants impart their phenotypic effect through functional effects on distal gene regulatory elements, such as enhancers. However, a challenge remains to pinpoint the causal variants that modulate these enhancers and to identify their effects on target genes in specific tissues. Recent studies have posited that regulatory variants are common, and may act in a pleiotropic manner to modulate expression across cell types^1,2^. Therefore, questions remain whether genetic associations are driven by single or multiple variants, if the phenotypic impact of causal variants is uniform or cell type-specific, and whether this regulation occurs across developmental stages or is confined to specific temporal windows^3–6^. The ability to characterize the genetic architecture of disease remains anchored in the need to develop and interpret comprehensive functional genomic maps in disease-relevant cell types. Here, we applied a suite of tools to generate genomic annotations that allow for the functional interpretation of GWAS loci associated with obesity.

GWAS meta-analyses for body mass index (BMI) have identified 97 independent loci associated with obesity, where the vast majority of these loci harbor causal variants that are predicted to be noncoding^7^. These noncoding BMI-associated variants are strongly enriched to lie within regions containing the brain and, to a lesser extent, adipose enhancers^7^. These two cell types are thought to be critical players in BMI maintenance, as they modulate energy intake in the form of hunger and satiety cues and control energy expenditure through central and peripheral circuitry. To functionally interpret obesity-associated loci, we systematically generated key genomic annotations in primary human adipocytes and human-induced pluripotent stem cell (iPSC)-derived hypothalamic neurons. To capture dynamic features of chromatin accessibility, gene expression, and long-range enhancer–promoter interactions, we assessed these parameters across the differentiation of iPSC-derived hypothalamic neurons and during the conversion of pre-adipocytes to mature white adipocytes. In addition, we identified a set of 2396 putatively causal variants in high linkage disequilibrium (LD) with the 97 BMI GWAS lead single nucleotide polymorphisms (SNPs) and determined the enhancer activity and allelic effects of each of these variants by performing a massively parallel reporter assay (MPRA) in brain and adipose cell lines.

Our MPRA data identified putatively causal enhancer-modulating variants (EMVars) with regulatory properties in adipose and/or neuronal cell lines. While we identified a single EMVar in some obesity-associated loci, the majority of loci contained multiple EMVars, demonstrating that the genetic architecture at GWAS loci is often complex. Assaying the regulatory landscapes of human adipocytes and hypothalamic neurons across developmental stages resulted in an increased overlap of functional annotations with EMVars, supporting evidence that a subset of functional variants have temporally restricted phenotypic effects in vivo. We synthesized these datasets to provide a ranking system for variant and target gene prioritization across 36 of the 97 GWAS loci to inform functional follow-up in each cell type. In addition, we characterized a particularly complex region of the genome on chromosome 16. This region harbors two independent GWAS loci that exhibited a dense network of cross-association in situ promoter capture Hi–C (cHi–C) connectivity and strong evidence of pleiotropy in our data. Altogether, our work utilizes high throughput technologies to shed light on the complexity of GWAS loci and applied a pipeline that can be used to prioritize GWAS target genes for functional follow-up for any heritable trait.

## Results

### Regulatory landscapes in obesity relevant cell types

**Fig. 1:**
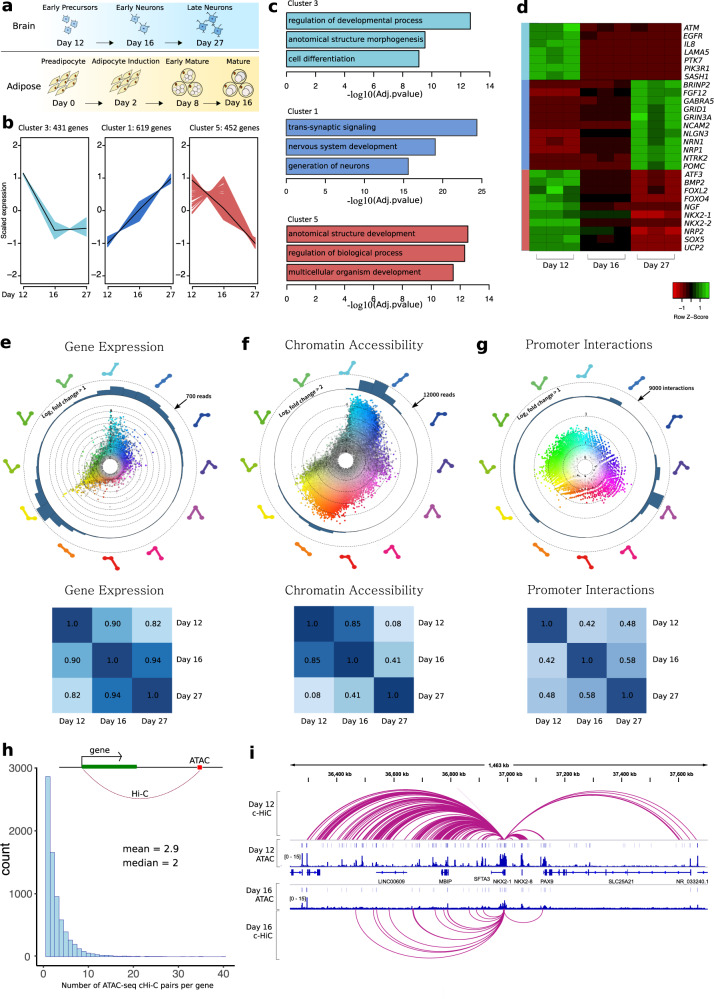
Characterizing hypothalamic differentiation using genomic annotations. **a** Time points for data collection. **b** Hypothalamic DEGs were grouped via fuzzy-c clustering and the top three clusters with the highest membership are illustrated. The number of genes in each cluster and scaled expression across the four differentiation time points are shown on each graph. **c** Significant Gene Ontology (GO) terms for the top three clusters. (Fisher’s Exact Test; FDR adjusted *p* values are presented). **d** A heatmap of gene expression depicting genes from each of the top three clusters that are members of the enriched GO terms. The leftmost colored bar indicates cluster membership and the columns are RNA-seq replicates. **e**–**g** HSV transformation of gene expression dynamics, ATAC-seq accessibility, and cHi–C interactions across differentiation. Each significant data point is categorized and colored based on the temporal pattern it displays shown by the guides on the periphery of each plot. The three nodes of each pattern represent day 12, day 16, and day 27 of neuronal differentiation. The distance of each point from the center of the circle represents maximum log_2_ fold change, and color transparency represents the relative number of reads for that data point. Below, heatmaps of Pearson’s *r* correlation coefficients estimate the overall similarity between time points. **h** On average, a promoter interacts with 2–3 ATAC-seq peaks via a c-HiC interaction across time (interactions and ATAC peaks were not required to be significant at the same time point) *n* = 7382 data points included. **i** View of significant cHi–C interactions emanating from the promoter of the *NKX2-1* gene, which is downregulated between differentiation days 12–16. ATAC-seq reads and significant ATAC-seq peaks at day 12 and day 16 are also shown.

To interpret obesity GWAS associations, we aimed to generate comprehensive maps of genome annotations in human hypothalamic neurons and adipocytes. Despite the prominence of hypothalamic neurons in obesity etiology little is known about the regulatory landscape of these cells, in part due to the challenge in obtaining them. To overcome this, we differentiated human iPSCs into mature hypothalamic neurons. We modulated sonic hedgehog (SHH), transforming growth factor β (TGFβ), and bone morphogenetic protein (BMP) signaling pathways to induce neuronal differentiation. After neuronal differentiation, we introduced brain-derived neurotrophic factor (BDNF) to promote the maturation of pro-opiomelanocortin (POMC) positive arcuate nucleus-type hypothalamic neurons. We collected cells at three time points representing early hypothalamic neuron precursors (D12), early immature (D16), and late (D27) mature hypothalamic neurons (Fig. 1a). These cells were then processed for cHi–C to elucidate putative enhancer–promoter interactions, ATAC-seq to identify open chromatin, and RNA-seq for global gene expression information. We also utilized non-immortalized Simpson–Golabi–Behmel syndrome (SGBS)^8^ human preadipocytes, which we differentiated to mature white adipocytes and collected during four key time points representing preadipocytes, differentiation induction, early mature adipocytes, and late mature adipocytes, respectively (Supplementary Figs. 1a, b and 3a). For each of the adipocyte time points, we also performed ATAC-seq, RNA-seq, and cHi–C^9–11^.

To characterize dynamic changes of these datasets across differentiation stages, we initially focused on identifying differentially expressed genes (DEGs) in both adipose and hypothalamic neurons. We used fuzzy-c means clustering to group DEGs into predominant patterns (Fig. 1b and Supplementary Fig. 2a, b). The three clusters with the highest membership scores are shown (Fig. 1b and Supplementary Fig. 3b–d). The first and third clusters (Cluster 3 and Cluster 5) encompassed genes that have relatively higher expression in early hypothalamic neurons and were enriched for genes involved in cellular differentiation and developmental processes. The second cluster (Cluster 1) represented genes that increased in expression during differentiation and were enriched for genes involved in synaptic signaling and neuron generation (Fig. 1b–d).

Using cHi–C, we identified 601,109–935,217 significant chromatin interactions per time point in adipose and 124,750–596,847 interactions in neurons, with a median interaction distance between 178 and 241 kb per library and approximately 20% promoter–promoter interactions (Supplementary Fig. 2c, d). The median fragment size for promoter-distal interactions was 604 base pairs, allowing us to map putative regulatory regions at very high resolution^12^. In support of these mapsʼ use for enhancer identification, we evaluated the promoter distal ends of the brain and adipose interactions and found they were enriched for cell type appropriate enhancer histone marks identified by ENCODE (H3K4me1 and H3K27ac), as well as for open chromatin (Supplementary Fig. 2e, f).

To derive comparisons across time and between datasets, we show all significant data points in hypothalamus and adipose using the Hue-Saturation-Value transformation (HSV)^13,14^ (Fig. 1e–g, Supplementary Fig. 3e–g). With HSV, gene expression, chromatin accessibility, and cHi–C promoter interactions can be visualized in a 360° space, where each significant data point is binned into a representative temporal pattern shown on the outside of the plot. We also performed a Pearson’s *r* correlation to evaluate relationships between time points. In neurons, gene expression and cHi–C were the least correlated between Day 12 and 16 (*r* *=* 0.90; RNA and *r* *=* 0.42; cHi–C) and reached an equilibrium in later differentiation (*r* *=* 0.94; RNA and *r* *=* 0.58; cHi–C). Conversely, the ATAC-seq data were the most correlated between Day 12 and 16 (*r* *=* 0.85) and changed more dramatically as differentiation continued (*r* *=* 0.41) (Fig. 1e–g). To evaluate whether changes in chromatin accessibility and/or cHi–C interactions correlate with gene expression changes at gene loci, we obtained a list of genes that were connected to at least one ATAC-seq peak via a significant cHi–C interaction in the hypothalamic differentiation (Fig. 1h). This list contained 7382 genes and on average each promoter interacted with 2–3 unique ATAC-seq peaks across the time-course (Fig. 1h). To test the functionality of these interactions, we grouped genes that were differentially expressed at any time point and compared changes in chromatin accessibility and cHi–C interaction strength to static genes. For genes upregulated between two-time points, we observed stronger interaction scores and more accessible chromatin compared to genes that were not differentially expressed. Downregulated genes also generally demonstrated stronger suppression of chromatin and interaction scores, supporting the use of these datasets to identify gene regulatory regions (Supplementary Fig. 2g). These analyses were also performed for the adipocyte differentiation and are presented in Supplementary Figs. 2a, c–g and 3a–i.

Using these data, we generated a high-resolution map of interactions between promoters and putative regulatory elements across several developmental stages in both adipose cells and iPSC-derived hypothalamic neurons. This is a critical first step for the overarching goal of obesity GWAS interpretation and allowed us to wire distant enhancers to promoters in a high-throughput manner (Fig. 1i).

### Identifying functional variation in obesity GWAS loci

**Fig. 2:**
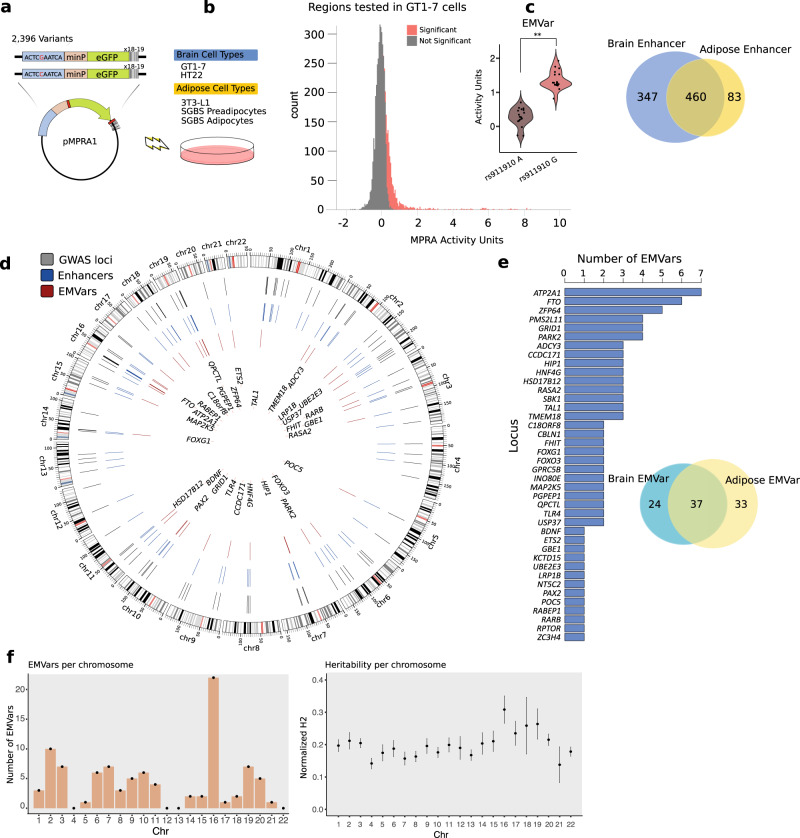
MPRA identifies enhancers and functional variants in obesity GWAS loci. **a** Variants were synthesized adjacent to 18–19 unique 10 bp DNA barcodes and cloned into the pMPRA1 vector. Constructs were transfected into five cell lines from the adipose and brain lineages (see Methods). **b** Average MPRA activity across the five replicates is shown for all tested regions in GT1–7 libraries. Significant GT1–7 enhancers (*q* *<* 0.05; one-sided Mann–Whitney *U* test) are colored red. An SNP was considered an EMVar if the variant significantly affected MPRA enhancer activity levels (**q = 4.2e−08; two-sided Mann–Whitney *U* test. **c** Venn diagram of MPRA enhancers significant in either cell type. **d** Circos plot of MPRA results. Grey lines within the circle represent the locations of GWAS associations, blue lines represent the locations of MPRA identified enhancers, and the red lines represent identified MPRA EMVars. Locus gene names (closest gene) are shown in the center. **e** Bar chart of significant EMVars per locus, along with a Venn diagram of EMVars called in either brain or adipose cell lines. **f** (left) Total number of significant EMVars identified per chromosome (*n* = 1 value per chromosome). (right) s-LDSC estimates from *n* = 1 BMI GWAS summary statistics of the heritability explained per chromosome normalized to the proportion of variants tested; data shown are percent heritability explained ± SEM (LD score regression with a block jackknife approach) (also see Supplementary Fig. 5).

A common mechanism by which noncoding variants lead to disease risk is expression modulation mediated by alterations in enhancer function^2^. In order to identify SNPs capable of affecting enhancer activity at GWAS loci, we employed an MPRA to test variants in high LD with lead SNPs identified in a recent BMI meta-analysis conducted by the GIANT consortium^7,15,16^. Candidate variants were defined as lead SNPs in 97 independent obesity GWAS loci and variants in high LD (MAF ≧ 5% CEU population, *r*^2^ > 0.8), for a total of 2396 variants. We chose to test all of these SNPs, rather than those within known annotations, in order to estimate the full scope of possible functional variants. We obtained 175-bp DNA fragments centered on each biallelic SNP, and each allele was synthesized alongside 18–19 unique 10 bp DNA barcodes, allowing for 18–19 measurements of enhancer activity for every allele. This resulted in a pool of 89,964 fragments that were cloned into the pMPRA1 vector^15^. We tested each region containing an SNP for enhancer modulating activity, as well as allele-specific differences in activity across three adipose cell types (SGBS preadipocytes, SGBS mature adipocytes, 3T3-L1 preadipocytes) and two neuronal cell types (GT1-7 and HT22 cells) (Fig. 2a, b). Across the GWAS loci, we identified 807 genomic regions in the brain and 543 genomic regions in adipose where at least one of the two alleles acted as an enhancer, and 460 regions were enhancers in both cell types (Fig. 2c). Of the enhancers, 94 harbored an EMVar, which conferred significant differences in enhancer activity between alleles (Fig. 2d). Compared to all tested regions, MPRA enhancers were enriched for ENCODE ChromHMM predicted active marks and depleted for inactive marks in adipose and brain tissues (Supplementary Fig. 4a). They were also more likely to overlap open chromatin and cHi–C interactions compared to tested regions without MPRA enhancer activity, supporting the potential of enhancer function for these regions in their native chromatin context (Supplementary Fig. 4b).

Because enhancers are made up of combinatorial transcription factor (TF) binding sites that can be up to 1 kb in length, we decided to validate enhancer activity for 24 unique ~1000 bp sized regions containing MPRA EMVars using luciferase assays in a brain and/or adipose cell line depending on where they were found to be significant using MPRA. We found that these longer DNA fragments resulted in the same enhancer activity call in luciferase assays for 28/43 (65%) of the conditions tested (Supplementary Fig. 4c). This indicates that while the size of the tested enhancer may affect activity, the calls were relatively consistent across different assay types and fragment sizes.

We next sought to illuminate the network of TFs putatively bound to these enhancers to understand potential biological processes regulated within these GWAS loci. We performed TF motif enrichment analysis within enhancer sequences identified from each of our MPRA cell types and found that MPRA enhancers at obesity GWAS loci were enriched for motifs for TFs that are involved in critical metabolic processes regulated in both brain and adipose. Multiple members of the *AP-1* complex family, as well as the *ATF* family, were identified. *AP-1* family members are upregulated during early adipogenesis and are critical for proper adipose formation, and also have the ability to regulate whole-body energy expenditure when modulated in the hypothalamus^17–20^. ATF factors, in particular, are important for adipogenesis and have also been shown to regulate thermogenic programs in the mouse hypothalamus through *Agrp* expression modulation^21–23^. Other TF motifs important for thermogenesis and glucose homeostasis were also enriched, including those for the thyroid hormone receptors, IRF3, ESRRA, and USF1/2^24–27^. Interestingly, TFs important for the maintenance of circadian rhythm in central or peripheral clocks such as CLOCK, bHLHe40 (DEC1), and BMAL1 were also enriched (Supplementary Fig. 5a, Supplementary Data 7)^28^.

In addition to identifying enhancers, the MPRA assay tests for variants capable of modulating enhancer activity (EMVars). Of the 94 EMVars, we found 61 brain EMVars and 70 adipose EMVars, and at least one EMVar was identified in 40/97 (41%) of tested GWAS loci. Surprisingly, 2/3 of these contained more than one EMVar (Fig. 2e). In addition, 37/94 (39%) of these variants affected enhancer activity in both cell types, an observation in line with the recent GTEx finding that the majority of expression quantitative trait loci (eQTLs) are not tissue-specific (Fig. 2e)^2^. This suggests that at each of these GWAS loci, multiple variants have the potential to contribute to expression variation. In addition, the effect of these variants may not be restricted to one obesity-relevant tissue.

The two loci that harbor the largest number of EMVars identified in our study were the *FTO* and *ATP2A1* obesity association regions, each representing strong and highly reproducible associations on chromosome 16 (Fig. 2e)^7^. Overall, we observed the largest number of EMVars mapping to chromosome 16. To investigate this further, we applied stratified LD score regression (s-LDSC)^29,30^ to BMI GWAS summary statistics to estimate heritability across chromosomes and confirmed that chromosome 16 contributes disproportionally to obesity heritability (Fig. 2f, Supplementary Fig. 5b, c)^31^. These data suggest that the strong heritability enrichment at chromosome 16 could be driven by an overabundance of functional variants, such as EMVars, that exist within chromosome 16 obesity GWAS loci.

### Assigning functional variants to target genes

**Fig. 3:**
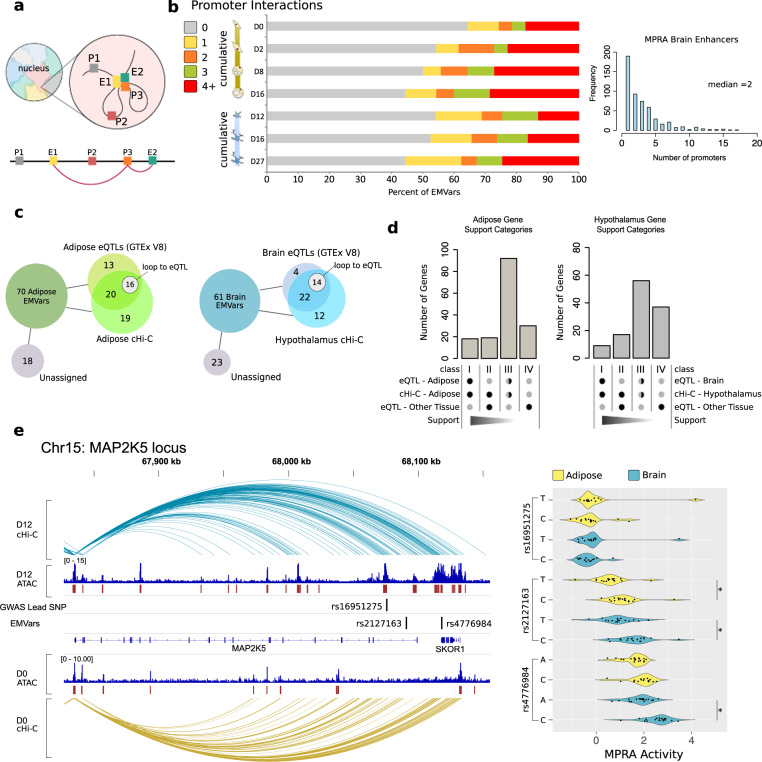
Integration of functional variants with genomic annotations prioritizes target genes. **a** cHi–C allows for the identification of physical connections between enhancers (E) and promoters (P) in nuclear space and are shown as arcs on the linear genome (depicted here in pink). **b** (left) Cumulative distribution of promoter interactions per EMVar across time in adipose and brain cells. (right) Bar plot showing the number of promoters that each Brain MPRA enhancer interacts with across all cHi–C replicates (does not include enhancers that do not interact with a promoter). **c** Diagram of EMVars that are either in cHi–C interactions and/or are GTEx eQTLs, and not assigned to a target gene with either method. **d** Genes were binned into classes based on strength of the evidence supporting them as a GWAS target gene (see Methods). A half shaded circle = eQTL or cHi–C support **e** *MAP2K5*, a class I gene, is shown here with the brain and adipose cHi–C interactions from its promoter. Average activity units for each EMVar barcode and lead SNP from this locus are shown in a violin plot in HT22 (blue) and 3T3-L1 cells (yellow). * Reached *q* *<* 0.05 in at least three out of five independent MPRA experiments; two-sided Mann–Whitney *U* test. Additional data are shown in Supplementary Data 6.

Having identified an array of regulatory elements and putatively functional variants in obesity GWAS loci, we next aimed to gain insights into the connections of these regulatory elements with their target genes in 3D genomic space using cHi–C (Fig. 3a). Understanding the configuration of these functional variants in respect to promoters in the nucleus will help identify target genes of the EMVar-containing enhancers and generate a list of prioritized genes for future mechanistic studies into their role in obesity etiology.

We intersected the time course cHi–C data generated in adipocytes and neuronal precursors with EMVar locations to identify interactions between EMVars and their target genes in a cell-type-specific manner. Having cHi–C data for multiple time points in both lineage differentiations allowed us to assign more EMVars to promoters (Fig. 3b). Interestingly, we observed that if an adipose EMVar, enhancer, ATAC-seq peak, or H3K27ac marked region participated in a cHi–C interaction, it contacted a median of three different promoters across the adipose cHi–C time-course. Similarly, brain EMVars and enhancers contacted a median of two promoters across the hypothalamic cHi–C time course (Fig. 3b). These data suggest pervasive pleiotropic regulation by these regulatory variants, which could affect gene expression in disparate tissues or in specific developmental stages. Our findings underscore the importance of assaying regulatory landscapes across development or under specific conditions, a finding recently corroborated by reports showing genomic annotations are subject to tissue and temporal specific regulation^3–5,32^.

To further assess the functionality of these long-range interactions and gain additional evidence for gene targets, we intersected EMVar-promoter interactions with eQTL information in adipose and brain cell types from GTEx^2^. For the brain, 26/61 EMVars were eQTLs for a gene in a GTEx(V8) brain cell type and 34/61 interacted with a promoter in at least one cHi–C library. Twenty-two EMVars participated in interactions with distant promoters and were eQTLs, while 14/22 were an eQTL for a gene they interacted with (Fig. 3c). We evaluated the intersection of adipose EMVars with our adipose differentiation cHi–C and GTEx subcutaneous or visceral adipose eQTLs in the same manner (Fig. 3c). Through the integration of these datasets across time, we were able to assign 38/61 (62%) of brain EMVars and 52/70 (74%) of adipose EMVars to at least one gene in the cell type where the EMVar was found to alter enhancer activity (Fig. 3c).

Integrating these annotations established gene expression patterns, identified regions of open chromatin, pinpointed enhancers harboring EMVars, and suggested target genes through long-range enhancer–promoter interactions in obesity-associated loci. To summarize this for each GWAS locus, we binned genes into four classes of supporting evidence for both adipose and brain based on degrees of supporting evidence for their involvement in obesity etiology (Fig. 3d, Supplementary Data 8). Identified Class I genes have functions with known relevance to BMI maintenance, such as cholesterol and steroid metabolism, food intake, fat mass, mitochondrial function, or leptin sensitivity^33–39^. There are also several genes with unknown functions or functions with unidentified relevance to a BMI phenotype.

As an example, we show that a brain EMVar (rs4776984) in the *MAP2K5* locus interacts with the Class I gene *MAP2K5* (Fig. 3e). This variant is a GTEx eQTL for *MAP2K5* in adipose, brain, and other cell types. This SNP-gene pair was previously tied to obesity risk in a study that identified this locus using eQTLs from the METSIM cohort and cHi–C data from primary human white adipose tissue^40^. We confirmed this SNP’s interaction in adipose and show that it also interacts with *MAP2K5* in iPSC-derived hypothalamic neurons (Fig. 3e). We also identified a second EMVar in this locus, rs2127163, which was an EMVar in both adipose and brain. This variant also interacted with and is an eQTL for, *MAP2K5* in both adipose and brain, suggesting that the association at this locus may be due to at least two different SNPs in independent enhancers regulating *MAP2K5* across these cell types (Fig. 3e). Our datasets thus allow us to tease apart loci with multiple potential causal variants and were not influenced by LD in the same manner as eQTL data alone.

### Extensive variant and gene-level pleiotropy within obesity-associated loci

**Fig. 4:**
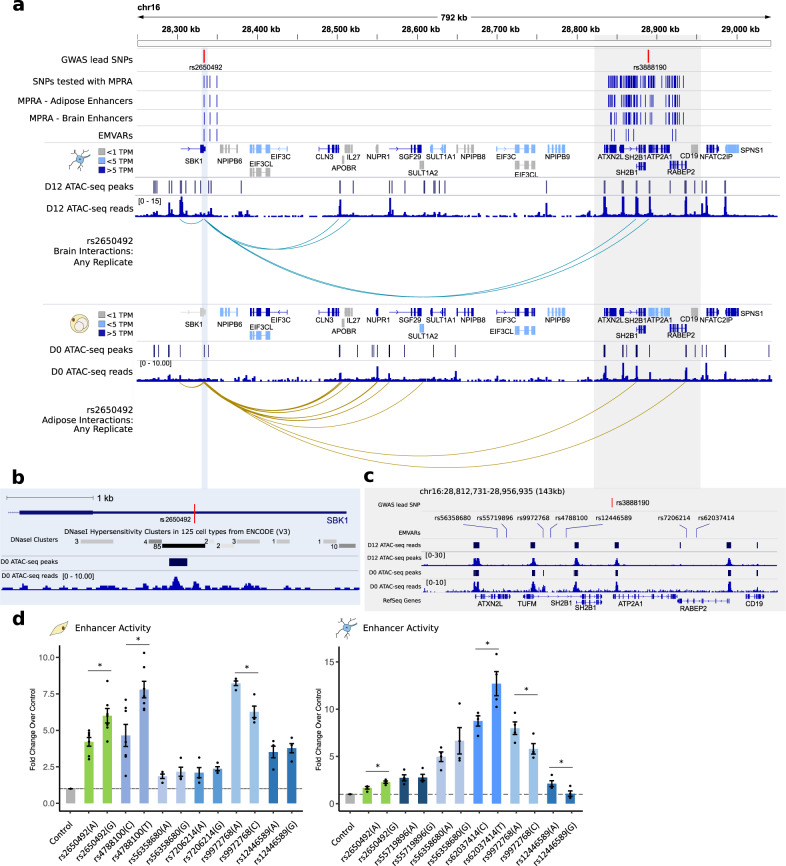
Two independent GWAS loci physically converge in nuclear space. **a** The locations of two lead GWAS variants separated by >0.5 Mb are depicted. cHi–C promoter interactions that encompass rs2650492 in the brain (blue) and adipose (yellow). b Location of rs2650492 within the 3′UTR of *SBK1*, along with significant DNAseI hypersensitivity clusters in 125 cell types from ENCODE. ATAC-seq peaks and read pileup from day 0 SGBS preadipocytes are also shown. **c** Location of all EMVars within the *ATP2A1* locus along with ATAC-seq peaks and read pileup from day 0 SGBS preadipocytes and day 12 early neuronal precursors. **d** Allele-specific luciferase assay results for EMVars in the HT22 neuronal cell line or SGBS preadipocytes. Fold change is calculated relative to the control sequence. (*n* = 4 independent HT22 experiments, *n* = 3 independent SGBS experiments for all variants with the exception of rs478100 and rs2650492 which had *n* = 7 independent experiments). *SGBS: rs2650492 *p* = 0.009; rs9972768 *p* = 0.03; rs4788100 *p* = 0.006; *HT22: rs2650492 *p* = 0.01; rs9972768 *p* = 0.04; rs62037414 *p* = 0.02; rs12446589 *p* = 0.04; two-tailed Student’s *t* test, data are presented as mean ± SEM.

While the integration of our functional genomics datasets was able to resolve regions such as the *MAP2K5* locus, where a single gene emerged as the likely target of the genetic association with obesity, we also uncovered unexpected complexities amongst other loci. As previously mentioned, despite its modest length, chromosome 16 had the strongest enrichment for obesity heritability of all human chromosomes and contained the largest number of EMVars in our study (Fig. 2e, f). This overrepresentation was partly due to a hotspot of EMVars within a 600 kb span on chromosome 16p11.2, which harbored two independent GWAS loci and 10 EMVars (Supplementary Fig. 6a, b). These two loci were the *SBK1* locus and *ATP2A1* locus, named for the gene closest to the lead variant. EMVars within these two regions formed an extensive network of long-range interactions with promoters within and between the reciprocal locus, suggesting that several of the genes in this megabase region may be co-regulated by a set of shared enhancers (Fig. 4a, Supplementary Fig. 6c). In addition, the EMVars within these two loci were eQTLs for, and physically interacted with, some of the largest number of genes in our dataset. Thus, a better understanding of these regions would shed light on a complex regulatory network within a GWAS-associated region and serve to test the ability of our datasets to uncover functional insights into obesity GWAS loci.

The first of these two regions harbored a lead SNP within the 3′UTR of *SBK1*. This SNP is not only associated with BMI, but also with other bodyweight phenotypes in the UK Biobank^41^. We identified three EMVars in this region. Two of these were neither eQTLs nor participated in cHi–C interactions and were discarded from the future investigation (Supplementary Fig. 6a). The third, rs2650492, is the lead SNP of this locus and is an eQTL for 18 nearby genes across GTEx cell types, including 5 in adipose and 7 in the brain. This EMVar also participated in cHi–C interactions with 13 genes, including those that extended beyond its locus into the neighboring *ATP2A1* locus over 500 kb away (Fig. 4a). In our MPRA datasets, the GWAS risk allele rs2650492-A decreased enhancer activity in both adipose and brain cell lines. This variant localized to open chromatin in our data as well as in a DNaseI cluster present in 85/125 ENCODE cell types (Fig. 4b).

The second region harbored a lead SNP, rs3888190, which mapped closest to the *ATP2A1* gene. SNPs in this locus have been associated with obesity in several studies, and the region harbors rare large copy number variations that lead to early onset obesity^42^. This region contained 7 EMVars, the largest number in our study, and 5 out of the 7 EMVars are in perfect LD with rs3888190 in the CEU population (Fig. 4c, Supplementary Fig. 6a). These variants are inherited together on a common European haplotype present at 32% frequency (Supplementary Fig. 6a). Five out of the seven alleles segregating on this risk haplotype decrease enhancer activity in the MPRAs. Three located between *SH2B1* and *TUFM* decrease enhancer activity, two located within *RABEP2* introns decrease enhancer activity, and two located within introns of *ATXN2L* increase enhancer activity (Supplementary Fig. 6b). Each SNP is an eQTL for nine genes in adipose and seven genes in the brain. Although eQTL status is confounded by linkage, each of these EMVars also contacted several promoters, indicating a capacity for independent regulation of multiple genes in the locus (Supplementary Fig. 6c).

Using luciferase assays, we confirmed EMVar enhancer activity for all *ATP2A1* locus EMVars and rs2650492 in either SGBS preadipocytes and/or HT22 brain cells depending on where they were active in MPRA (six actives in adipose and six active in the brain). We validated that 3/6 adipose EMVars and 4/6 brain EMVars had allelic effects detectable by luciferase assay. Both rs2650492 and rs9972768 were confirmed to affect enhancer activity in both cell types (Fig. 4d). Overall, these data suggested that multiple functional variants within distinct enhancers are present at this obesity-associated locus, each with the potential to regulate multiple genes, thus contradicting the canonical model of a single casual variant affecting a single target gene. The pleiotropic regulatory impact on different genes would result in complex molecular signals emanating from multiple genes that, together, participate in disease etiology. Given that 27/40 (67.5%) of EMVar containing loci had more than putatively functional variant (Fig. 2d), our data suggests that this complex connectivity will be a common feature of disease-associated loci, and is not simply an oddity of this region.

### Investigating enhancer activity under unique cellular and developmental contexts

**Fig. 5:**
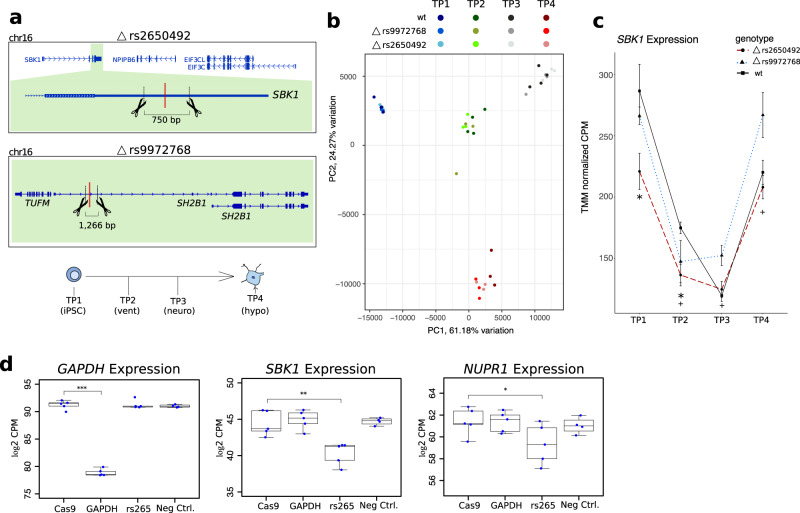
Functional variants in GWAS loci regulate multiple local genes in *cis*. **a** Genomic regions targeted by CRISPR–cas9 editing machinery in iPSCs. (top) A 750 bp region within the 3′UTR of *SBK1* containing rs2650492 was targeted for deletion, and (middle) a second 1.3 kb region in between *TUFM* and *SH2B1* surrounding rs9972768 was deleted in an independent line. (bottom) iPSCs were differentiated to the hypothalamic lineage and collected at four-time points for RNA-seq. **b** PCA plot showing all genotypes and time points collected for RNA-seq during differentiation to hypothalamic neuronal precursors. **c** Plot of TMM normalized counts per million (CPM) for *SBK1* across time points. * rs2650992 deletion lines TP1 *q* = 0.001, TP2 *q* = 0.004, ^+^rs9972768 deletion lines TP2 *q* = 0.04, TP3 *q* = 0.002, TP4: *q* = 0.016; data are shown as mean ± SD (*n* = 3 clones rs2650492 deletion lines; rs9972768 deletion lines; WT lines TP4 and *n* = 4 clones WT lines TP1–3). **d** CRISPRi of enhancer containing rs2650492 in HEK293t cells. The expression after removal of batch effects for significantly differentially expressed *cis*-genes identified in RNA-seq analysis across CRISPRi conditions in HEK293t cells. left panel ***p = 5.1e−33, middle panel **p = 1.0e−4, right panel *p = 3.1e−3 (*n* = 5 independent experiments Cas9; GAPDH; rs265 and *n* = 4 independent experiments Neg Ctrl). boxplot center line, median; box limits, upper and lower quartiles; whiskers, 1.5× interquartile range.

To provide additional evidence for a functional connection between the *ATP2A1* and *SBK1* GWAS loci on chromosome 16, we used CRISPR-cas9 editing to delete one EMVar-containing enhancer from each locus. Out of the ten EMVars identified across the two loci, rs2650492 and rs9972768 were the most supported for causality because they were in open chromatin, they participated in many long-range interactions with distal genes, and they were eQTLs for several genes within the megabase encompassing these regions. We generated polyclonal cell lines where we deleted the regions harboring rs2650492 or rs9972768 in a human iPSC cell homozygous for the non-risk haplotypes at both GWAS loci (Fig. 5a). We used the BrainSpan atlas^43^ of gene expression in addition to expression data collected from our hypothalamic differentiation and determined that all genes within this megabase region are expressed uniformly across early development except *NUPR1*, which is lowly expressed until post conception. We, therefore, chose to assay the effects of these variants during early hypothalamic development. Four independent deletion clones from each line were then differentiated to the hypothalamic lineage. Cells were collected at four-time points representing key early developmental stages: iPSCs, ventralized cells, neuronal precursors, and hypothalamic precursors (Fig. 5a, b). Total RNA was extracted from each line at every time point and RNA-seq was performed to identify genes affected by these enhancer deletions.

Although these two SNPs map to enhancers separated by over half a megabase, we found that these deletions independently affected the expression of a single common gene, *SBK1*, and the effect was temporally restricted (Fig. 5c). Both enhancers were critical for proper *SBK1* expression during TP2 (ventralization), as *SBK1* expression was reduced in both lines at this stage. The convergent regulation of *SBK1* by variants from two independent GWAS loci supports the cross-locus cHi–C interactions that we observed, as well as the eQTL effect of both rs2640492 and rs9972768 variants on *SBK1* expression.

Looking globally at gene expression patterns in these two lines, we observed extensive sharing of a large number of DEGs between the two enhancer deletions. DEG sharing increased dramatically between the ventralization (TP2) and neuronal precursor timepoints (TP3), suggesting these two enhancers converge to regulate an early driver gene that, when misregulated, leads to a cascade of gene expression changes (Supplementary Fig. 7a–c). Although the function of human *SBK1* is unknown, its zebrafish homolog, *Bsk146*, is critical for early neuronal development. Upon *Bsk146* knockdown, zebrafish embryos exhibited changes to the midbrain–hindbrain boundary, enlarged hindbrain ventricles, and had small eyes^44^. Mice lacking *Sbk1* have also been shown to exhibit an abnormal neurological phenotype^45^. In both of our enhancer deletion lines, Gene Ontology terms for neural development genes were enriched within DEGs, supporting a conserved role for *SBK1* in this process (Supplementary Data 10).

While our data in neural lineage cells demonstrated that enhancers within the two independent GWAS loci regulate *SBK1*, it did not address the evidence of pleiotropy suggested by the many cHi–C interactions. Therefore, we evaluated the ability of the enhancer harboring rs2650492 to regulate additional genes under a secondary context. We targeted the rs2650492 EMVar, the promoter of *GAPDH* as a positive control, and a negative control inactive region within the chromosome 16 locus using CRISPRi machinery in HEK293t cells (Supplementary Fig. 7d). We FACS sorted cells containing the Cas9 alone, or Cas9 and CRISPRi guides, to select for cells that were transfected with the CRISPRi components. We extracted total RNA from these cells and performed RNA-seq to look for the downregulation of genes within the two loci. We observed a significant expression decrease for one distal gene in the locus, *NUPR1*, which was very lowly expressed throughout our neuronal RNA-seq time course (<1 TPM all stages) but is moderately expressed in HEK293t and in adipose (Fig. 5d). The rs2540492 EMVar was an eQTL for *NUPR1* in several GTEx tissues, and in our data formed frequent long-range cHi–C interactions with *NUPR1* in our adipocyte differentiation but not in the brain. We also observed a significant decrease in *SBK1* expression after targeting rs2650492 in HEK293t cells. However, because rs2650492 maps within the 3′UTR of *SBK1*, we could not rule out that the change in expression we detect may be due to the possible hindrance of *SBK1* transcription or reduction in stability due to the recruitment of CRISPRi machinery to the 3′UTR^46^.

Altogether, our data show that at least two MPRA identified EMVars in this complex locus is within independent enhancers that regulate at least one common gene. One of these enhancers, which harbors the lead SNP of the *SBK1* locus, is additionally capable of regulating a second gene in an alternate cell type. The pleiotropic physiological effects stemming from these tissue and temporal regulatory specificities may play a role in the molecular etiology of obesity risk, and highlight complex considerations in the functional experiments that will attempt to better understand the mechanisms underlying GWAS associations to disease susceptibility.

## Discussion

In this work, we generated comprehensive regulatory maps in human adipose and hypothalamic neurons, which lack complete genomic annotations despite their prominence in disease etiology. We profiled these cells across several differentiation stages to catalog chromatin accessibility, expression patterns, and cHi–C enhancer–promoter interactions which, together, aid in the interpretation of candidate causal non-coding variants at obesity-associated loci. Recent work suggest that regulatory variants associated with human phenotypes may impart their effect during temporally restricted windows, which would be missed in functional assays of a single developmental time point or environmental perturbation^3–5,32^. Thus, integrating cHi–C data from multiple time points along with GTEx eQTL data allowed us to better assign putative gene targets to functional variants and to provide an evidence-based ranking approach for genes within obesity GWAS loci.

Interestingly, we frequently identified multiple SNPs within a locus capable of modulating enhancer activity across two obesity-relevant cell types. This suggests a previously underappreciated degree of allelic and tissue heterogeneity. Recent reports support this notion, with strongly powered and densely genotyped GWAS identifying independent signals within the same association to suggest the existence of multiple causal variants within a single loci^47^. But few studies to date have investigated how regions containing multiple causal variants affect gene expression across tissues and developmental time points. With its increasingly larger dataset, the GTEx consortium has recently been able to address patterns of tissue specificity in eQTLs and found a high level of eQTL effect sharing between tissues^2^. This indicates functional variants may have pleiotropic effects across tissues as frequently, or more frequently than tissue-specific effects.

While we have provided evidence that in a number of obesity-associated loci there are multiple functional variants potentially regulating gene expression, we are not able to formally ascertain the impact of those variants on gene expression. It would be interesting to test this hypothesis using statistical fine-mapping, which explicitly identifies all potential causal variants in trait-associated loci^48^. This is made difficult, however, by the lack of individual-level GWAS data. When only summary statistics of GWAS are available, as in our case, fine-mapping methods will have to rely on LD information derived from external reference samples. Potential mismatch of GWAS sample LD and reference LD may greatly increase false-positive findings. Multiple simulation studies have shown that essentially with GWAS from large meta-analysis, it is only possible to reliably identify single causal variants in trait-associated loci^49,50^. Another challenge of using fine-mapping to establish allelic heterogeneity is that all current fine-mapping methods impose “sparsity”, i.e., the number of causal variants in a locus is small. So, if one variant is detected as putatively causal, any variant in high LD would be unlikely to be found as an additional causal variant. In our results, multiple functional variants in a locus may be in high LD (e.g., the *ATP2A1* locus, see Fig. 4c), so even if there are multiple causal variants in a locus, the current fine-mapping tools will not be able to detect them as separate signals. The dissection of the locus on chromosome 16 that we performed in the current work (Fig. 5) as well as the obesity-associated *FTO* locus^51^ support our model of multiple variants acting on distinct enhancers at restricted temporal windows. Future work will determine if these insights are extended to the other loci and variants we report here. A second limitation to this work involves the use of immortalized cell lines. Although we used non-immortalized karyotype normal cells to generate RNA-seq, ATAC-seq, and cHi–C profiles across differentiation, we were limited due to the requirement for high transferability and millions of cells for the MPRA assays. Therefore, we had to rely on immortalized cell lines for some of this enhancer activity and enhancer modulating estimates.

We identified 23% (22/94) of EMVars on chromosome 16 alone. This, and the obesity heritability enrichment of chromosome 16, suggests that this chromosome could harbor an overabundance of obesity-relevant genes. A complex region in 16p11.2 harbors three independent GWAS loci within a megabase range. Within this interval, multiple local segmental duplications in the great ape’s lineage have resulted in new genes and transcripts in the human genome^52^. These repeat regions leave this region vulnerable to structural variants, resulting in deletions and duplications^53^, where deletions lead to highly penetrant forms of obesity^42,54,55^. The gene(s) and mechanisms mediating BMI phenotypes in this region remain a focus of investigation. This region emerged from our datasets due to the high complexity of long-range interactions and patterns of eQTL sharing among many genes across adipose and brain. Our data demonstrate that these loci harbor multiple functional SNPs within enhancers that independently regulate *SBK1* early in neuronal differentiation. In addition, we demonstrated that rs2650492, a GWAS lead variant, regulates at least one other gene, *NUPR1*, in a secondary cellular context. While the profiling of cells carrying deletions of enhancers was done in cells harboring independent genomic deletions using CRISPR technology, the editing was carried out in a single individual, raising the possibility that some of the effects observed might not be similar in a different genetic background.

Other critical modulators of the obesity phenotype exist within the *ATP2A1* locus, such as *SH2B1*, a gene involved in leptin and insulin signaling^37,38^. This gene and others were eGenes that physically connected to multiple EMVars. It is likely that investigation of these EMVars under other conditions or developmental stages would uncover further examples of pleiotropy in this locus. It has yet to be elucidated whether many or only a subset of genes, modulated within this region are capable of contributing to obesity risk.

Altogether, our work raises the possibility that the underlying genetic architecture of individual loci associated with obesity may often involve allelic heterogeneity, where multiple variants in distinct regulatory elements impart expression effects on the gene(s) across tissues during uniform or restricted temporal windows. Whether the complexities we uncovered here are a rule or exception for loci in variant-to-function studies has yet to be addressed and will require careful investigation of causal genetic variation at GWAS loci under multiple cell types and temporal conditions.

## Methods

### SGBS culture and differentiation

*SGBS cells were a gift from Dr. Martin Wabitsch*. SGBS were maintained and differentiated as previously described^8,56^. Briefly, cells were grown in DMEM/F12 (1:1) media (Life Technologies #11330-032) with 10% fetal bovine serum, 1% penicillin–streptomycin solution (10,000 U/ml; Gibco #15140122), 8 mg/ml Panthotenic Acid, and 8 mg/ml Biotin (Sigma; #B4639). Cells were allowed to grow to 70–80% confluency before splitting 1:3 with 0.25% Trypsin–EDTA (Gibco; #25200056). To differentiate, cells were split into 6-well plates, allowed to reach 100% confluency over two days. After maintenance of confluency, day 0 cells were harvested, and the remaining cells were washed twice with 1× phosphate-buffered saline (PBS) and exposed to Quick-Diff media. Quick-Diff media consists of serum-free DMEM/F12 media supplemented with 0.01 mg/ml human transferrin (Sigma #T2252), 20 nM human insulin, 100 nM cortisol, and 0.2 nM Triiodothyronine. Day 2 cells were harvested two days after the addition of quick-diff media. Cells were incubated for a total of 4 days in quick-diff media before 3FC media was added. The 3FC media consists of serum-free DMEM/F12 media supplemented with 0.01 mg/ml human transferrin (Sigma #T2252), 20 nM human insulin, 100 nM cortisol, 0.2 nM Triiodothyronine, 25 nM dexamethasone, 250 µM 3-isobutyl-1-methylxanthine (IBMX), and 2 µM rosiglitazone. Mature adipocytes are maintained on 3FC media until collection.

*Human iPSCs*. We used one iPSC line throughout this manuscript (Yoruban—line NA19101) which was generated as part of a previously published study^57^. The iPSCs were derived from lymphoblastoid cells, they are able to differentiate into all three germ layers and display a normal karyotype. The feeder-independent iPSCs were maintained at 70% confluence on Matrigel hESC-qualified Matrix (354277, Corning, Bedford, MA, USA). Cells were cultured in complete mTeSR1 media (Stemcell #85850) supplemented with 1% Penicillin–Streptomycin (10,000 U/ml; Gibco #15140122) at 37 °C in 5% CO_2_. Cells were passaged when they were ~70% confluent with enzyme-free dissociation solution (30 mM NaCl, 0.5 mM EDTA, 1× PBS minus Magnesium and Calcium) and maintained in mTESR1 with 10 μM Y-27632 dihydrochloride (Abcam #ab120129) for up to 24 h. The medium was replaced daily.

*Human hypothalamic neuron differentiation*. iPSCs were differentiated into hypothalamic arcuate-like neurons, as previously described by Wang et al.^58^. Briefly, the SHH signaling pathway was activated and TGFβ and BMP signaling were inhibited, followed by inhibition of the NOTCH pathway, leading to neuronal induction, ventralization, and hypothalamic differentiation. After differentiation, the brain-derived neurotrophic factor (R&D Bioscience #248*‐*BD) was introduced to promote neuronal maturation of pro-opiomelanocortin (POMC) neurons. The neurons were then maintained in this medium supplemented with BDNF and B27 supplement (Thermo Fisher #17504044). Cells were collected at different time points and processed for in situ promoter capture HiC (cHi–C), total RNA extraction, and ATAC-seq.

### Timecourse RNA-seq

*Adipose*. At days 0, 2, 8, and 16 of differentiation 1 million, SGBS cells were collected and frozen per replicate with three technical replicates per time point. When all time points were collected, cells were lysed with a 20*g* needle, and total RNA was extracted using the RNeasy kit (Qiagen #74104). RNA quality was assessed on an agarose gel. RNA-seq libraries were generated from 1 μg of total RNA following the Illumina.

*TruSeqRNA Sample Preparation V2 guide and 15 cycles of polymerase chain reaction (PCR) amplification*. Libraries were sequenced on an Illumina HiSeq 4000 machine. *Neurons*: At days 12, 16, and 27 of differentiation approximately 1 million cells were collected and frozen per replicate with three technical replicates per time point. When all time points were collected, cells were lysed with a 20*g* needle, and total RNA was extracted using the RNeasy kit (Qiagen #74104). RNA quality was assessed on an Agilent Bioanalyzer and only samples with RIN > 8 were retained. RNA-seq libraries were generated from 500 ng of total RNA following the NEB Next Ultra II Directional RNA-seq kit and 10 cycles of PCR amplification. *Analysis*: Analysis included three technical replicates per time point. Gene-level read counts were quantified in each technical replicate at each time point directly using salmon (v0.7.2), correcting for sequence-specific bias and using a gene list derived from GENCODE release grch37.v19. For individual gene expression, read counts per gene were converted into transcripts-per-million (TPM) to account for gene length and library size. For the purposes of HSV visualization, gene counts were converted to TPM to normalize for transcript length and then adjusted for library size using the trimmed mean of *M*-values (TMM) normalization. Mean TPM was calculated at each time point and all genes with mean log_2_(TPM) < 1 at any time point were removed from further analysis.

### Timecourse clustering

Gene-level read counts were quantified in each technical replicate at each time point directly using salmon(v0.7.2), correcting for sequence-specific bias and using a gene list derived from GENCODE release grch37.v19. Gene-level read counts were transformed into counts-per-million (CPM) and any gene with CPM < 1 in more than three samples across all time points was removed from further analysis. The data were normalized to account for library size using TMM normalization. Linear models testing pairwise differential expression between any two-time points were fit using limma(v3.48.1). and tested using a moderated *t*-test accounting for mean-variance dependence and increased dispersion in limma. All genes with significant differential expression between any two-time points were included in the clustering analysis. Raw gene-level counts from salmon were normalized to account for transcript length and scaled to account for differences in gene expression across genes. Fuzzy c-means clustering was performed in R using the e1071 package. A gene was assigned to the cluster for which it had the highest membership if (1) its membership score was above 0.3 for the averaged replicates and (2) above 0.2 for each individual replicate. The top three clusters were defined by the highest average membership score.

### HSV plots

All HSV analyses are developed from code originally published in Siersbaek et al.^14^. Value (*V*) indicates the maximum log_2_(counts in TPM or CPM) for a given gene at any time point, and so is defined as

1 $$V=\,{{\max }}({C}_{t})$$

Saturation (S) indicates the maximal fold change between any time points and is defined as

2 $$S=1-\frac{{{{\min }}}_{t}({C}_{t})}{V}$$

Hue (H) indicates the pattern of change in gene expression across time and is defined as

3 $$H=60* \left(2+\frac{{C}_{O}+{C}_{2}-{C}_{16}-V}{V* S}\right)* \frac{{C}_{2}-{C}_{O}}{|{C}_{2}-{C}_{O}|}$$

For visualization purposes, the values of *V* and *S* were scaled between 0 and 1 based on rank. MPRA cell lines culture and transfection: HT22 (Millipore Sigma): Cells were maintained in DMEM (Gibco # 11995-065) supplemented with 10% fetal bovine serum (FBS) and 1% penicillin–streptomycin solution (10,000 U/ml; Gibco #15140122) at 37 °C in 5% CO_2_. We plated 250,000 cells into wells of 6-well plates and transfected 1 day later with Lipofectamine LTX & Plus reagent (Invitrogen; #15338100) when 60–70% confluent. 3T3-L1 (ATCC): Cells were maintained in DMEM (Gibco # 11995-065) supplemented with 10% FBS, 1% penicillin–streptomycin solution (10,000 U/ml; Gibco #15140122), 0.8 mg/ml Biotin (Sigma; #B4639) and 0.8 mg/ml Panthotenic Acid at 37 °C in 5% CO_2_. We plated 20,000 cells into wells of 6-well plates and transfected 2.5 days later with Lipofectamine LTX & Plus reagent (Invitrogen; #15338100) when 30–50% confluent. GT1-7 (Millipore Sigma): Cells were maintained in High Glucose DMEM (Gibco # 10313-021) supplemented with 10% FBS, 1% penicillin–streptomycin solution (10,000 U/ml; Gibco #15140122) and 1× Glutamax (Gibco #35050061) at 37 °C in 5% CO_2_. We plated 750,000 cells into wells of 6-well plates and transfected the next day with Lipofectamine LTX & Plus reagent (Invitrogen; #15338100) when 60–70% confluent. SGBS Preadipocyte (D0): 30,000 SGBS preadipocyte cells were plated into 24-well plates and transfected with Polyplus jetPEI DNA transfection reagent (Polyplus; #101-10N) when 50% confluent. These cells were collected 48 h later for RNA processing. *SGBS Adipocyte (D8)*: Cells were plated as described above for differentiation. On differentiation day 8, cells were transfected with Lipofectamine LTX & Plus reagent (Invitrogen; #15338100) and collected on differentiation day 10.

### ATAC-seq

We harvested 100,000 fresh SGBS cells or neurons per time point with two technical replicates. ATAC-seq libraries for each cell type were generated according to the protocol outlined in Buenrostro et al.^9^. The cells were lysed, centrifuged, and frozen at −80 °C until all time points were collected. Final processing of all pellets was performed together. Transposed DNA fragments were PCR amplified using 5–7 PCR cycles. PCR cycle number was determined using qPCR reactions where the additional cycle numbers were those that corresponded to the inflection point of the qPCR curve. Peak Calling: ATAC-seq reads were trimmed to remove Nextera adapters using cutadapt (v8.25) and aligned to the genome using Bowtie2 (v2.3.2). All reads mapping to the mitochondrial genome were removed from further analyses. Peak calling was performed using macs2 (v2.1.1.20160309) using no model and an extension size of 200. Significant peaks were considered those which survived FDR correction (*q* < 0.05). *HSV analysis*: The union set of significant peaks across time points was obtained, and peaks of a uniform length of 1 kb were obtained by centering around the summit of the highest peak per peak locus in the union set. The counts per time point mapping to these 1 kb union peaks were obtained and transformed to log_2_(CPM) format, normalizing by library size (defined as a total number of reads in peaks per sample). Hue, saturation, and value were calculated using the same equations as with gene expression, using normalized log_2_(CPM) values as input.

### In situ promoter capture HiC

In situ promoter capture HiC was performed and analyzed as previously described^10,11^. Briefly, 5 million SGBS cells or neurons per replicate were harvested, counted, and crosslinked using a final 1% (v/v) concentration of formaldehyde (Sigma #252549) for 10 min at room temperature while rocking. This reaction was quenched with 0.25 M Glycine (Sigma #G8898) to a final concentration of 0.2 M for 5 min and washed with 1× PBS. Cells were frozen in liquid nitrogen and stored at −80 °C until ready for the next stage of in situ promoter-capture Hi–C processing. Each differentiation time point has two technical replicates and was sequenced on a full lane of an Illumina Hiseq 4000 machine to achieve sufficient read depth for interaction calling. *Data analysis*: In situ promoter capture Hi–C reads were aligned to the genome using Bowtie2(v2.3.2) and technical artifacts were removed using HiCUP (v0.5.9). Significant interactions were detected over a background model of null expectation using CHiCAGO (v1.2.0). Only interactions with a CHiCAGO score > 5 at any time point were included in downstream analyses. Trans-chromosomal interactions and interactions between loci greater than 1 megabase apart were filtered from further analysis. Counts were normalized by library size using (TMM) normalization and transformed into CPM. Hue, saturation, and value were calculated using the same equations as with gene expression, using normalized CPM values as input.

### Massively parallel reporter assay

Lead variants for BMI were taken from the 2015 GIANT consortium meta-analysis, which identified 97 independent significant loci. We searched 1000 genomes phase 3 genotypes (ftp.1000genomes.ebi.ac.uk/vol1/ftp/release/20130502) for the 97 GWAS lead SNPs and obtained all CEU SNPs (Utah residents with Northern and Western European ancestry from the CEPH collection) within 50 kb and with *r*^2^ > 0.8 with a lead SNP. We only retained biallelic SNPs with MAF ≧ 5% (2396 in total). Using these variants, MPRA oligo design was performed as previously described with modifications^15^. We synthesized 230 bp long DNA fragments as seen below using a 100,000 oligonucleotide Agilent array.

5′-ACTGGCCGCTTCACTG-enh-GGTACCTCTAGA-barcode-AGATCGGAAGAGCGTCG-3′ Each enh region was 175 base pairs of endogenous DNA context surrounding one of the biallelic 2346 SNPs. Each allele of each biallelic variant was synthesized beside 18–19 unique 10 bp DNA barcodes. Barcodes were randomly generated using a series of A, C, T, or Gs that did not contain three or more of the same base in a row and did not create Kpn1 or Xba1 restriction enzyme sites. We also later determined that barcodes should not end with the sequence “TCT”, because it creates a restriction enzyme site with the beginning of the second constant region and they will thus be lost. Upon receipt, this fragment pool was dissolved in 100 µl of nuclease-free water and PCR amplified using the Micellula DNA emulsion and Purification Kit (EURx #E3600-01) in order to reduce amplification bias of particular oligos over others. This PCR adds homology arms onto the oligos and allowed us to use Gibson Assembly Master Mix (NEB # E2611S) to clone these oligos into a linearized pMPRA1 vector (addgene #49349) that was cut open using the SfiI restriction enzyme. This backbone + oligo insert vector was then linearized using a Kpn1 and Xba1 double digest and a 60 bp truncated eGFP containing a minimal promoter and spacer sequence (141 bp in size) was ligated in between the enh fragments and barcodes using T4 DNA ligase (NEB; # B0202S) at a 1:10 ratio of insert to vector and incubated at 16 °C overnight. The resulting plasmid library was linearized a final time using Kpn1 and size selected for vectors containing all inserts (oligos + eGFP insert) on a 1% agarose gel. This is then religated using T4 DNA ligase and transformed until enough final plasmid is produced for all transfections. At each cloning and transformation, step complexity must be maintained, so we counted colony forming units (CFUs) after each transformation and aimed to attain at least 100 million CFUs. To ensure the best transformation, all reactions were cleaned up with the Minelute PCR purification kit (Qiagen; # 28004) and then further cleaned on a Millipore drop dialysis membrane (Millipore; #VSWP02500) for an hour before the transformation. Cells were transformed into MegaX DH10B T1R electrocompetent bacteria (Invitrogen #C640003) and allowed to grow for only 7–9 h after recovery to ensure the likelihood of getting high CFUs without bias towards particular constructs. Once the final constructs were produced, they were transfected into GT1–7 cells (6 replicates), 3T3-L1 cells (7 replicates), HT22 cells (5 replicates), SGBS Day 0 cells (6 replicates), and SGBS Day 8 cells (5 replicates) as described above. Enough cells were transfected to achieve a minimum of 10 million transfected cells per replicate with transfection efficiency estimated using a GFP control plasmid. Cells were collected 48 h after transfection and flash-frozen in liquid nitrogen until all replicates were collected. A replicate was considered “technical” if they were transfected with the same batch of DNA on different days with different cell passages. A replicate was considered “biological” if the input DNA library was separately cloned from the beginning from our Agilent oligonucleotides. MPRA experiments were designed to have 2–4 technical replicates for each of two biological replicates. After transfection, cells were lysed using a 20*g* needle, and RNA was extracted using Qiagen RNeasy mini kit. RNA quality was assessed on a 1% agarose gel. mRNA was isolated from total RNA using Invitrogen Dynabeads (ThermoFisher #61006) and then treated with Promega RQ1 DNAse (Promega; M6101) for 1.5 hours at 37 °C with an enzyme boost halfway through the reaction. The isolated and DNA plasmid-depleted mRNA is then cleaned up with the Qiagen RNeasy mini kit and quantified using the Promega QuantiFluor RNA system (Promega #E3310). Importantly, enrichment of RNA transcripts emanating from our MPRA plasmid compared to MPRA DNA plasmid contamination is assessed at this point using qPCR primers targeted to the eGFP. To do this, 250 ng of mRNA is converted to cDNA while 250 ng of mRNA is run through the cDNA reaction without reverse transcriptase (RT). We then perform a qPCR to determine enrichment of eGFP transcripts between RT(+) and RT(−) samples. We set a threshold of a minimum of eight CT enrichment between DNA and RNA as a quality control check. All remaining mRNA is then converted to cDNA using Superscript III RT. cDNA is treated with RNAse A (Invitrogen #12091-021) and RNAse T1 (ThermoFisher #EN0541) for 1 h and then cleaned with the Qiagen Minelute PCR purification kit. Totally, 50 ng of cDNA is then used as a PCR template for the final Illumina multiplexing primers. All available cDNA should be amplified. Two 50 ng reactions of Input DNA (DNA used as transfection material) must also be PCR amplified with Illumina multiplexing primers at this point. Libraries were amplified with 10–11 PCR cycles using Q5 Hot Start High-Fidelity 2× Master Mix (NEB #M0494S) and pooled before clean up using Agencourt AMPure XP beads (0.6x + 1.2x double cleanup; Beckman Coulter; #A63882). Library quality was assessed using the Agilent DNA 1000 bioanalyzer chip (Agilent; #G2938-90014), where a single sharp peak of around 250 bp is expected. Samples can then be sent for paired-end NGS sequencing. A 25% PhiX genome spike must be added to each sequencing run due to low complexity. *Data analysis*: Barcode reads must be converted to the reverse complement before they can be matched with known barcodes. We required sequenced barcodes to be exact matches with expected barcode sequences. Count data is then analyzed for significance as previously described^16^. In essence, lowly expressed barcodes were removed and enhancer activity was determined from the remaining normalized counts using the following equation:

Enhancer activity = log2 ((output CPM) − (input CPM))(4)

The activity was then quantile normalized and enhancer *p* values were calculated using a one-sided Mann–Whitney *U* test in R using the wilcox.test function. *p* Values were corrected for multiple testing using the p.adjust function, method = “fdr” from the stats package(v3.6.2) in R. All regions where at least one allele was determined to be a significant enhancer were then tested for enhancer modulating effects using a two-sided Mann–Whitney *U* test in R with *p* values adjusted for multiple testing using the p.adjust function. Enhancer modulating variants were retained for downstream analyses if they were significant in half of all technical replicates or both biological MPRA replicates.

### MPRA enhancer validation luciferase assays

Twenty-one regions containing at least one SNP that had an allele in a significant enhancer in either HT22 and/or 3T3-L1 cells were chosen for validation. These regions were PCRed from genomic DNA using Q5 Hot Start 2× Master Mix (NEB #M0494S) and were designed to be ~1 kb in size. Each region was cloned into the pGL4.23 luciferase vector containing firefly Luciferase and was tested for luciferase activity via co-transfection with renilla luciferase at a ratio of (1:50) in both 3T3-L1 cells and HT22 cells. Alleles were determined through Sanger sequencing. Renilla and firefly luciferase fluorescence was measured on a Promega GloMax microplate reader using the Dual-Luciferase Reporter Assay System (Promega #E1910). Firefly luciferase measurements were normalized to renilla measurements and then fold change over a control DNA region was calculated to determine enhancer activity.

### ATP2A1-SBK1 EMVar luciferase assays

In order to test allele-specific enhancer activity, IDT gBlocks^™^ were ordered containing each allele of each variant. Each SNP tested was centered and surrounded by 470 base pairs of native genomic context and 15 bp of homology on each side to the pGL4.23 luciferase vector. These gblocks were then cloned into the pGL4.23 luciferase vector using Gibson assembly. rs4788100 (C) and (T) gblocks were surrounded by 295 bp of native genomic context, rs62037414 (C) and (T) gblocks were surrounded by 451 bp of genomic context, and rs55719896 (G) and (A) gblocks were surrounded by 469 bp of genomic context due to synthesis constraints. Luciferase assays were conducted either in SGBS preadipocytes or HT22 cells as described above.

### LDSC partitioned heritability analysis

Heritability per chromosome was calculated via LD score regression analysis using the ldsc command-line tool (v1.0.0) using Locke et al. 2012 BMI GWAS summary statistics and Yengo et al.^59^ GMI GWAS summary statistics downloaded from the GIANT consortium. Briefly, bim files from 1000 Genomes Phase 1 were downloaded and annotation files were created for each chromosome where the chromosome was treated as a binary annotation. LD scores were then computed from these annotation files for input into partitioned heritability analysis. Summary statistics were filtered to contain only HapMap3 variants as advised.

### Transcription factor motif analysis

All regions identified to be significant enhancers were included in this analysis. Regions were expanded to be 175 bp (size of enhancers tested in MPRA) and then if two regions overlapped they were then merged so they would not become overrepresented in the analysis. The program findMotifsGenome.pl from HOMER^60^(v4.8.3) was then used in addition to the -size flag to identify motifs that were overrepresented in significant MPRA enhancers from each cell line. These were compared to size and base composition matched set of background sequences computed by HOMER to determine significance and *p* value. All *p* values from each cell line are included in Supplementary data 7.

### Calling MPRA EMVAR interactions with promoters

MPRA EMVars were considered to interact with a promoter if the distal end of the promoter interaction came within 1 kb of the single base pair SNP location. EMVar SNP location and cHi–C BEDPE files were overlapped using the BEDtools (v2.27.1)^61^ pairToBed function. These variants were not required to overlap other annotations, such as ATAC-seq.

### Gene support classes

To develop gene level support for each GWAS locus, we first binned EMVars into their respective loci. For Class I genes, we required an EMVar to interact with that gene and the EMVar must be a GTEx eQTL for that gene in the appropriate cell type (GTEx adipose tissues for adipose EMVars and GTEx Brain tissues for Brain EMVars). Class II genes interact with an EMVar in the appropriate cHi–C dataset and the EMVar is an eQTL for that gene in cell types other than the appropriate one. Class III genes were those that interacted with an EMVar in the appropriate cHi–C libraries or were an eGene for this SNP in the correct cell type. Class IV genes were eGenes for these EMVars in other cell types. In addition, we only included genes in this analysis that were expressed > 1 TPM in at least 1-time point in their respective cell type from our RNA-seq (all genes with their classes from both cell types are shown in Supplementary Data 8).

### CRISPR–cas9 editing of iPSC cells

iPSCs were grown in complete mTeSR1 media (Stemcell #85850) supplemented with 1% penicillin–streptomycin (10,000 U/ml; Gibco #15140122) on Matrigel-coated dishes (Corning #354277) at 37 °C in 5% CO_2_. Fluorescently tagged crRNA-Atto550 and cas9 protein were purchased from IDT. Guides were designed using IDT software in order to maximize cutting efficacy and minimize off-target cleavage using their RNP system. Guide RNAs were selected such that if they mapped elsewhere in the genome, there were at least two base pair mismatches to mitigate the chance of off-target effects. Two guides were designed per region in order to delete the enhancers from their endogenous context. IDT crRNA and tracrRNA were complexed according to the manufacturer’s instructions. On the day of transfection, 50 µM of each guide and cas9 protein were combined and incubated at room temperature for 20 min to form RNPs. Each guide was complexed with cas9 in individual reactions. Totally, 900 k of human iPSC cells were harvested and nucleofected using a Lonza nucleofector 2b device with the program A23 and the two RNP complexes. These cells were plated into one 22 cm^2^ flask. Once recovered, 40,000 cells were split into a 100 cm^2^ flask to achieve single-cell colonies. Colonies were then picked and transferred into 48-well plates to grow independently. Colonies were screened for the presence of homozygous deletion bands using PCR. Homozygous deletion colonies were grown, transfected, split, and treated identically to wild-type (WT) cells in order to mitigate RNA-seq batch effects for the differentiation. Cells remained frozen until all timepoints were collected. RNA was then extracted using the Qiagen RNeasy Kit and RNA quality was assessed via an Agilent Bioanalyzer RNA Chip. One microgram of RNA was used for input into RNA-seq. RNA-seq was performed using the NEB Next Ultra II Directional RNA-seq kit. Data Analysis: Reads were mapped and gene counts were quantified with STAR (v2.5.1a). Counts were filtered to retain autosomal genes and exclude lowly expressed genes (<1 CPM). Data clustered well by time-point and genotype so no batch effect correction was necessary. *p* Values were identified using glmQLFTest() from edgeR (3.28.1). *p* Values were FDR adjusted genome-wide using p.adjust. PCR primers and guides are in Supplementary Data 11.

### HEK293t CRISPRi

HEK293t cells (ATCC) were maintained in DMEM (Gibco # 11995-065) supplemented with 10% FBS and 1% penicillin–streptomycin (10,000 U/ml; Gibco #15140122) solution at 37 °C in 5% CO_2_. Four guide RNAs were designed for each targeted region by CHOPCHOP or MIT’s guide design tool based on maximizing cutting efficiency, minimizing off-targets, and closest proximity to the region of interest. Guide sequences are provided in the Supplementary Data. Guides were then individually cloned using golden gate methodology into the guide vector upstream of an eGFP gene. Cells were transfected into HEK293t cells at 50–70% confluency using Lipofectamine LTX with the 4 guide vectors and dCas9 vector (dCas9–KRAB upstream of BFP fluorophore) at a ratio of three parts Cas9 to one part guide, where each individual guide was added in equal amounts to additional guides for that region. After 48 hours, double-positive GFP and BFP fluorescing cells were collected into culture media via FACs sorting, spun down, and frozen. Sorting was performed on a BD FACS Aria Fusion 5–18 using BD FACSDiva 8.0.1 software. In the case of the Cas9 only control population, BFP single positive cells were sorted out of the population via FACS and frozen. This was repeated 4–5 times to have technical replicates. RNA was extracted using the Qiagen RNeasy RNA mini extraction kit and 500 ng of RNA was used as input for RNA-seq. RNA-seq was performed using the NEB Next Ultra II Directional RNA-seq kit. *Data analysis*: Reads were mapped and gene counts were quantified with STAR (v2.5.1a). Counts were filtered to retain autosomal genes and exclude lowly expressed genes (<1 CPM). PC1 clearly associated with the sorting batch, so the sorting batch was added as a covariate into the final linear model. *p* Values were identified using glmQLFTest() from edgeR. Because of the very small effect sizes of CRISPRi in non-coding regions, *cis*-genes within the SBK1-ATP2A1 loci were considered for final significance testing. This list included all protein-coding genes within these loci passing the expression threshold as well as *GAPDH* (14 genes total). Raw *p* values from these genes were Bonferroni corrected to get adjusted *p* values (*p* *<* 0.05/14 genes). Only genes passing the Bonferroni significance threshold were considered significantly affected by the CRISPRi perturbations.

### Reporting summary

Further information on research design is available in the Nature Research Reporting Summary linked to this article.

## Supplementary information

Supplementary Information

Description of Additional Supplementary Files

Supplementary Data 1-11

Reporting Summary

## Data Availability

All high throughput sequencing data that support the findings of this study have been deposited in https://www.ebi.ac.uk/arrayexpress/ with the following accession codes: rs9972768 and rs2650492 deletion RNA-seq (E-MTAB-10464), MPRA (E-MTAB-10463), ATAC-seq (E-MTAB-10462), time course RNA-seq (E-MTAB-10461), rs2650492 CRISPRi RNA-seq (E-MTAB-10460), and in situ promoter capture Hi–C (E- MTAB-10488). Publically downloaded data used in this paper ChIP-seq data can be downloaded from: https://egg2.wustl.edu/roadmap/data/byFileType/peaks/consolidated/narrowPeak/E063-H3K4me1.narrowPeak.gz https://egg2.wustl.edu/roadmap/data/byFileType/peaks/consolidated/narrowPeak/ E063-H3K27ac.narrowPeak.gz https://egg2.wustl.edu/roadmap/data/byFileType/peaks/consolidated/narrowPeak/E081-H3K4me1.narrowPeak.gz ChromHMM 15 state predictions can be downloaded from https://egg2.wustl.edu/roadmap/data/byFileType/chromhmmSegmentations/ChmmModels/coreMarks/jointModel/final/E063_15_coreMarks_mnemonics.bed https://egg2.wustl.edu/roadmap/data/byFileType/chromhmmSegmentations/ChmmModels/coreMarks/jointModel/final/E081_15_coreMarks_mnemonics.bed

## Code availability

Code will be made available upon request.

## References

[1] Hormozdiari F (2017). Widespread allelic heterogeneity in complex traits. Am. J. Hum. Genet..

[2] Consortium T (2020). Gte. The GTEx Consortium atlas of genetic regulatory effects across human tissues. Science.

[3] Strober BJ (2019). Dynamic genetic regulation of gene expression during cellular differentiation. Science.

[4] Claussnitzer M (2015). FTO Obesity Variant Circuitry and Adipocyte Browning in Humans. N. Engl. J. Med..

[5] Beagan JA (2020). Three-dimensional genome restructuring across timescales of activity-induced neuronal gene expression. Nat. Neurosci..

[6] Smemo S (2014). Obesity-associated variants within FTO form long-range functional connections with IRX3. Nature.

[7] Locke AE (2015). Genetic studies of body mass index yield new insights for obesity biology. Nature.

[8] Fischer-Posovszky P, Newell FS, Wabitsch M, Tornqvist HE (2008). Human SGBS cells—a unique tool for studies of human fat cell biology. Obes. Facts.

[9] Buenrostro JD, Wu B, Chang HY, Greenleaf WJ (2015). ATAC-seq: a method for assaying chromatin accessibility genome-wide. Curr. Protoc. Mol. Biol..

[10] Rao SSP (2014). A 3D map of the human genome at kilobase resolution reveals principles of chromatin looping. Cell.

[11] Montefiori LE (2018). A promoter interaction map for cardiovascular disease genetics. eLife.

[12] Cairns, J. et al. CHiCAGO: robust detection of DNA looping interactions in capture Hi-C data. *Genome Biol*. **17**, 127 (2016). 10.1186/s13059-016-0992-2 PMC4908757 27306882

[13] Stavreva, D. A. et al. Dynamics of chromatin accessibility and long-range interactions in response to glucocorticoid pulsing. *Genome Res*. 10.1101/gr.184168.114 (2015). 10.1101/gr.184168.114 PMC4448681 25677181

[14] Siersbæk R (2017). Dynamic rewiring of promoter-anchored chromatin loops during adipocyte differentiation. Mol. Cell.

[15] Melnikov A (2012). Systematic dissection and optimization of inducible enhancers in human cells using a massively parallel reporter assay. Nat. Biotechnol..

[16] Ulirsch JC (2016). Systematic functional dissection of common genetic variation affecting red blood. Cell Traits Cell.

[17] Stephens JM, Butts MD, Pekala PH (1992). Regulation of transcription factor mRNA accumulation during 3T3-L1 preadipocyte differentiation by tumour necrosis factor-alpha. J. Mol. Endocrinol..

[18] Distel RJ, Ro HS, Rosen BS, Groves DL, Spiegelman BM (1987). Nucleoprotein complexes that regulate gene expression in adipocyte differentiation: direct participation of c-fos. Cell.

[19] White UA, Stephens JM (2010). Transcriptional factors that promote formation of white adipose tissue. Mol. Cell. Endocrinol..

[20] Idelevich A (2018). Neuronal hypothalamic regulation of body metabolism and bone density is galanin dependent. J. Clin. Investig..

[21] Cheng C-F (2019). Adipocyte browning and resistance to obesity in mice is induced by expression of ATF3. Commun. Biol..

[22] Liu Y (2019). The transcription factor ATF7 controls adipocyte differentiation and thermogenic gene programming. iScience.

[23] Lee Y-S (2013). Hypothalamic ATF3 is involved in regulating glucose and energy metabolism in mice. Diabetologia.

[24] Pelletier P, Gauthier K, Sideleva O, Samarut J, Silva JE (2008). Mice lacking the thyroid hormone receptor-alpha gene spend more energy in thermogenesis, burn more fat, and are less sensitive to high-fat diet-induced obesity. Endocrinology.

[25] Dahle MK, Taskén K, Taskén KA (2002). USF2 inhibits C/EBP-mediated transcriptional regulation of the RIIβ subunit of cAMP-dependent protein kinase. BMC Mol. Biol..

[26] Laurila P-P (2016). USF1 deficiency activates brown adipose tissue and improves cardiometabolic health. Sci. Transl. Med..

[27] Shimomura, K. et al. Usf1, a suppressor of the circadian Clock mutant, reveals the nature of the DNA-binding of the CLOCK:BMAL1 complex in mice. *eLife* **2**, e00426 (2013). 10.7554/eLife.00426 PMC3622178 23580255

[28] Honma S (2002). Dec1 and Dec2 are regulators of the mammalian molecular clock. Nature.

[29] Finucane HK (2015). Partitioning heritability by functional annotation using genome-wide association summary statistics. Nat. Genet..

[30] Bulik-Sullivan BK (2015). LD Score regression distinguishes confounding from polygenicity in genome-wide association studies. Nat. Genet..

[31] Shi H, Kichaev G, Pasaniuc B (2016). Contrasting the genetic architecture of 30 complex traits from summary association data. Am. J. Hum. Genet..

[32] Calderon D (2019). Landscape of stimulation-responsive chromatin across diverse human immune cells. Nat. Genet..

[33] Praggastis M (2015). A murine Niemann-Pick C1 I1061T knock-in model recapitulates the pathological features of the most prevalent human disease allele. J. Neurosci..

[34] Rantakari P (2010). Hydroxysteroid (17β) dehydrogenase 12 is essential for mouse organogenesis and embryonic survival. Endocrinology.

[35] Gamero-Villarroel C (2017). Influence of TFAP2B and KCTD15 genetic variability on personality dimensions in anorexia and bulimia nervosa. Brain Behav..

[36] Williams, M. J. et al. Obesity-linked homologues TfAP-2 and Twz establish meal frequency in *Drosophila melanogaster*. *PLoS Genet*. **10**, e1004499 (2014). 10.1371/journal.pgen.1004499 PMC4154645 25187989

[37] Doche ME (2012). Human SH2B1 mutations are associated with maladaptive behaviors and obesity. J. Clin. Investig..

[38] Ren D (2007). Neuronal SH2B1 is essential for controlling energy and glucose homeostasis. J. Clin. Investig..

[39] Hershkovitz T (2019). A novel TUFM homozygous variant in a child with mitochondrial cardiomyopathy expands the phenotype of combined oxidative phosphorylation deficiency 4. J. Hum. Genet..

[40] Pan DZ (2018). Integration of human adipocyte chromosomal interactions with adipose gene expression prioritizes obesity-related genes from GWAS. Nat. Commun..

[41] Sudlow C (2015). UK Biobank: an open access resource for identifying the causes of a wide range of complex diseases of middle and old age. PLOS Med..

[42] Jacquemont S (2011). Mirror extreme BMI phenotypes associated with gene dosage at the chromosome 16p11.2 locus. Nature.

[43] Miller JA (2014). Transcriptional landscape of the prenatal human brain. Nature.

[44] Chou C-M (2006). Expression and characterization of a brain-specific protein kinase BSK146 from zebrafish. Biochem. Biophys. Res. Commun..

[45] Blake JA (2017). Mouse Genome Database (MGD)-2017: community knowledge resource for the laboratory mouse. Nucleic Acids Res..

[46] Qi LS (2013). Repurposing CRISPR as an RNA-guided platform for sequence-specific control of gene expression. Cell.

[47] Mahajan A (2018). Fine-mapping of an expanded set of type 2 diabetes loci to single-variant resolution using high-density imputation and islet-specific epigenome maps. Nat. Genet..

[48] Schaid DJ, Chen W, Larson NB (2018). From genome-wide associations to candidate causal variants by statistical fine-mapping. Nat. Rev. Genet..

[49] Benner C (2017). Prospects of fine-mapping trait-associated genomic regions by using summary statistics from genome-wide association studies. Am. J. Hum. Genet..

[50] Weissbrod O (2020). Functionally informed fine-mapping and polygenic localization of complex trait heritability. Nat. Genet..

[51] Sobreira DR (2021). Extensive pleiotropism and allelic heterogeneity mediate metabolic effects of IRX3 and IRX5. Science.

[52] Lander ES (2001). Initial sequencing and analysis of the human genome. Nature.

[53] Antonacci F (2010). A large and complex structural polymorphism at 16p12.1 underlies microdeletion disease risk. Nat. Genet..

[54] Bochukova EG (2010). Large, rare chromosomal deletions associated with severe early-onset obesity. Nature.

[55] González JR (2014). A common 16p11.2 inversion underlies the joint susceptibility to asthma and obesity. Am. J. Hum. Genet..

[56] Wabitsch M (2001). Characterization of a human preadipocyte cell strain with high capacity for adipose differentiation. Int. J. Obes. Relat. Metab. Disord. J. Int. Assoc. Study Obes..

[57] Banovich NE (2018). Impact of regulatory variation across human iPSCs and differentiated cells. Genome Res..

[58] Wang L (2015). Differentiation of hypothalamic-like neurons from human pluripotent stem cells. J. Clin. Investig..

[59] Yengo L (2018). Meta-analysis of genome-wide association studies for height and body mass index in 700000 individuals of European ancestry. Hum. Mol. Genet..

[60] Heinz S (2010). Simple combinations of lineage-determining transcription factors prime cis-regulatory elements required for macrophage and B cell identities. Mol. Cell.

[61] Quinlan AR, Hall IM (2010). BEDTools: a flexible suite of utilities for comparing genomic features. Bioinformatics.

