# Supplementary material for: A functional genomics pipeline identifies pleiotropy and cross-tissue effects within obesity-associated GWAS loci
Source: Nat Commun. 2021 Sep 6;12:5253. doi: 10.1038/s41467-021-25614-3 (PMC8421397; doi:10.1038/s41467-021-25614-3)

Supplementary Figure Legends

Supplementary Figure 1: Differentiation markers

a) Representative images during differentiation of SGBS adipocytes along with expression for several marker genes during the four time points ($n = 3$ replicates, data shown are mean expression estimates ± SD). b) Representative images during differentiation of iPSC derived hypothalamic neurons along with expression of several marker genes during the three time points ($n = 3$ replicates, data shown are mean expression estimates ± SD).

Supplementary Figure 2: Features of functional annotations

a) Six fuzzy–c means clusters were identified for adipose and b) hypothalamic DEGs from the RNA-seq time course. The number of genes comprising each cluster, along with scaled expression across the time points is shown. c) Overview of median interaction length and number of interactions per time-point in the replicate-merged cHi-C datasets. Number of ATAC-seq peaks from the replicate-merged time points. d) Bar plot depicting proportion of promoter-promoter interactions in merged cHi-C libraries. e,f) The promoter-distal ends of interactions are enriched for functional ChIP-seq peaks and ATAC-seq peaks compared to a distribution of randomly chosen, number-matched set of non-promoter MboI fragments within mappable genomic regions ($n = 100$ iterations). The fold change of the observed overlap over 100 randomized sets is presented. ChIP-seq datasets were obtained from Adipose Nuclei (E063) and Fetal Brain (E081) repositories from the Roadmap Epigenomics project. (* $p < 0.05$; two-sided Z-test) Data shown as the average of the 100 randomizations ± SD g) Genes were binned based on upregulation or downregulation across each time point. Plotted are the changes in interaction score or normalized ATAC-seq reads for ATAC peaks connected through these genes via a significant cHi-C peak between each time point. boxplot center line, median; box limits, upper and lower quartiles; whiskers, 1.5x interquartile range; outliers not shown *$p < 0.05$; two-sided Mann-Whitney U test. Adipose values for each bin (Day 0-2) n = 2,205 up, n = 3,998 down, n = 26,752 not DE; (Day 2-8) n = 653 up, n = 717 down, n = 31,575 not DE, (Day 8-16) n = 545 up, n = 849 down, n = 31,551 not DE; Neuron values for each bin (Day 12-16) n = 1,588 up, n = 2,457 down, n = 20,726 not DE; (Day 16-27) n = 1,495 up, n = 363 down, n = 22,913 not DE.

Supplementary Figure 3: Characterizing Adipocyte Differentiation using Genomic Annotations

a) Time points for data collection b) Adipose DEGs were grouped via fuzzy-c means clustering and the top three clusters with highest membership scores are illustrated. The number of genes in each cluster and scaled expression across the four differentiation time points is depicted. c) Significant KEGG pathway terms identified using Enrichr for the top three clusters (Fisher's Exact Test; p values are adjusted for multiple tests). d) Heatmap of gene expression depicting genes from each of the top three clusters that are members of the enriched KEGG pathway terms. The leftmost colored bar indicates cluster membership and each column is an RNA-seq replicate. e-g) HSV transformation of expressed genes, ATAC-seq peaks, and cHi-C interactions across differentiation. The three nodes of each pattern represent day 0, day 2, and day 16 of adipose differentiation. The distance of each point from the center of the circle represents maximum $\log_2$ fold change, and color transparency represents the relative number of reads for that data point. Below, heatmaps of Pearson's r

correlation coefficients estimate overall similarity between time points. h) On average, a promoter interacts with 3-4 ATAC-seq peaks via a cHi-C interaction across time (interactions and ATAC peaks were not required to be significant at the same time point). i) View of cHi-C interactions from the promoter of the *IRS2* gene, which becomes upregulated between differentiation days 0-2. ATAC-seq reads and peaks from day 0 and day 2 are also shown.

Supplementary Figure 4: MPRA enhancer activity is supported

a) MPRA enhancers are enriched for Epigenome Roadmap's 15 state ChromHMM functional marks in adipose nuclei or fetal brain compared to all tested variants b) MPRA enhancers are enriched for presence in cHi-C interactions, number of interactions per enhancer, and open chromatin compared to non-significant regions. (*$p < 0.05$; two-sided Student's t-test) c) Luciferase assay results for ~1 kb sized regions containing an EMVar. Regions were chosen at random, and represent a full spectrum of MPRA enhancer p values. Because of this, non-EMVar rs1026737 and rs10000940 were included because they had very low and high enhancer p values, respectively. If the allele that was captured was not a significant enhancer, the result is colored with a grey background. Interestingly, for the rs4430895 region, we were able to clone both alleles, and although neither allele was an enhancer using the luciferase assay, the allele predicted to be stronger with MPRA had higher Luc2 expression compared to the weak allele. $n = 9$ independent replicates for all constructs except $n = 12$ independent replicates for rs2836753_G, rs1800437_G, rs6091542_T + rs6096971_A + rs6096972_G, rs3800231_T, rs41303827_G, rs843812_A, rs7146955_A, rs7030846_T, rs1406256_C, rs78575557_A and rs794364_A tested in in 3T3-L1 cells. boxplot center line, median; box limits, upper and lower quartiles; whiskers, 1.5x interquartile range. rs794364_A_3T3 p = 4.8e-3; rs794364_A_HT22 p = 4.7e-3; rs7146955_A_HT22 p = 1.1e-3; rs56358680_A_HT22 p = 2.3e-4; rs56358680_A_3T3 p = 1.7e-3; rs4788211_A_HT22 p = 8.5e-5; rs4788211_A_3T3 p = 3.8e-6; rs2382538_A_3T3 p = 7.0e-4; rs2382538_A_HT22 p = 1.96e-8; rs1800437_G_3T3 p = .02; rs1800437_G_HT22 p = 7.72e-6; rs13204087_C + rs13220728_T_3T3 p = 1.8e-4; rs13204087_C + rs13220728_T_HT22 p = 8.4e-5; rs116735807_T_3T3 p = 3.3e-3; rs116735807_T_HT22 p = 2.3e-10; rs1026737_T_HT22 p = 2.0e-4; rs1026737_T_3T3 p = 2.6e-7; rs3800231_T_3T3 p = 7.0e-3

Supplementary Figure 5: Transcription factors in obesity associated loci and additional analysis of chromosome 16

a) (left) Position weight matrices and identifiers for enriched transcription factor motifs from HOMER. Each motif was enriched in either MPRA adipose or brain enhancers (HOMER adjusted p value < 0.05; Binomial Test). (right) Transcription factors are connected to a BMI relevant phenotype with a line if these factors play a role in that biological process (significant in both brain and adipose = grey circle, significant in adipose = yellow, and significant in brain = blue). b) s-LDSC estimated proportion of total heritability explained per chromosome is depicted along with heritability enrichment values. data shown are percent heritability explained ± SEM (LD score regression with a block jackknife approach) c) Number of EMVars compared to the number of variants tested with MPRA stratified per chromosome.

Supplementary Figure 6: Haplotype information and SNP specific interactions for *ATP2A1* locus EMVars

a)(left) Summary information for all 10 EMVars identified in both the *SBK1* and *ATP2A1* loci. Two SNPs in the *SBK1* region were neither eQTLs nor did they participate in cHi-C interactions and were thus removed from future consideration. (right) Allele frequencies and haplotype information in the CEU population for all EMVars in the *ATP2A1* locus (LDhap tool: https://ldlink.nci.nih.gov). The lead risk variant, rs3888190-A, is outlined in blue. b) MPRA allele specific activity levels for EMVars within the *ATP2A1* locus and *SBK1* locus in adipose or brain libraries. Data presented are the average activity for each barcode across independent experiments . *reached $q < 0.05$ in at least half of all independent experiments or in both biological replicates. exact *p* values per replicate are presented in the Supplementary Tables; two-sided Mann-Whitney U test. c) Promoter interactions stemming from each EMVar in the *ATP2A1* locus at any time point in both brain and adipose cells. Location of variant is indicated by a red line (b,c) Adipose cHi-C data=yellow, Neuronal cHi-C data = blue

Supplementary Figure 7: A high level of sharing exists between independent enhancer deletions

a) Venn Diagrams depicting numbers of significantly differentially expressed genes between enhancer deletions and WT cells at each time point. The Jaccard Index is a representation of sharing on a scale of 0-1, where 1 is complete sharing and 0 is no sharing. b) Heatmap showing the Pearson *r* correlations between $\log_2$FC of all expressed autosomal genes in the genome for the two enhancer deletion lines compared to WT cells. c) Gene Ontology enrichments for differentially expressed genes that were significantly upregulated or downregulated at two time points during the differentiation. Corresponding volcano plots show directionality and numbers of the differentially expressed genes. (Fishers Exact Test; FDR adjusted *p* values are presented) d) Locations of CRISPRi guides for each condition. Guides were designed to target the 3'UTR of *SBK1*, the promoter of *GAPDH* as a positive control, and a region downstream of *TUFM* as a negative control. Cells were transfected and isolated via FACS based on the presence of either the Cas9 expressing BFP plasmid (BV421-A) and/or the GFP expressing guide plasmid (FITC-A). The lower left FACS panel represents cells that were not expressing BFP or GFP as a gating control. The lower middle FACS panel represents BFP positive cells and the lower right panel represents double positive BFP/GFP expressing cells.

Supplementary Figure 1

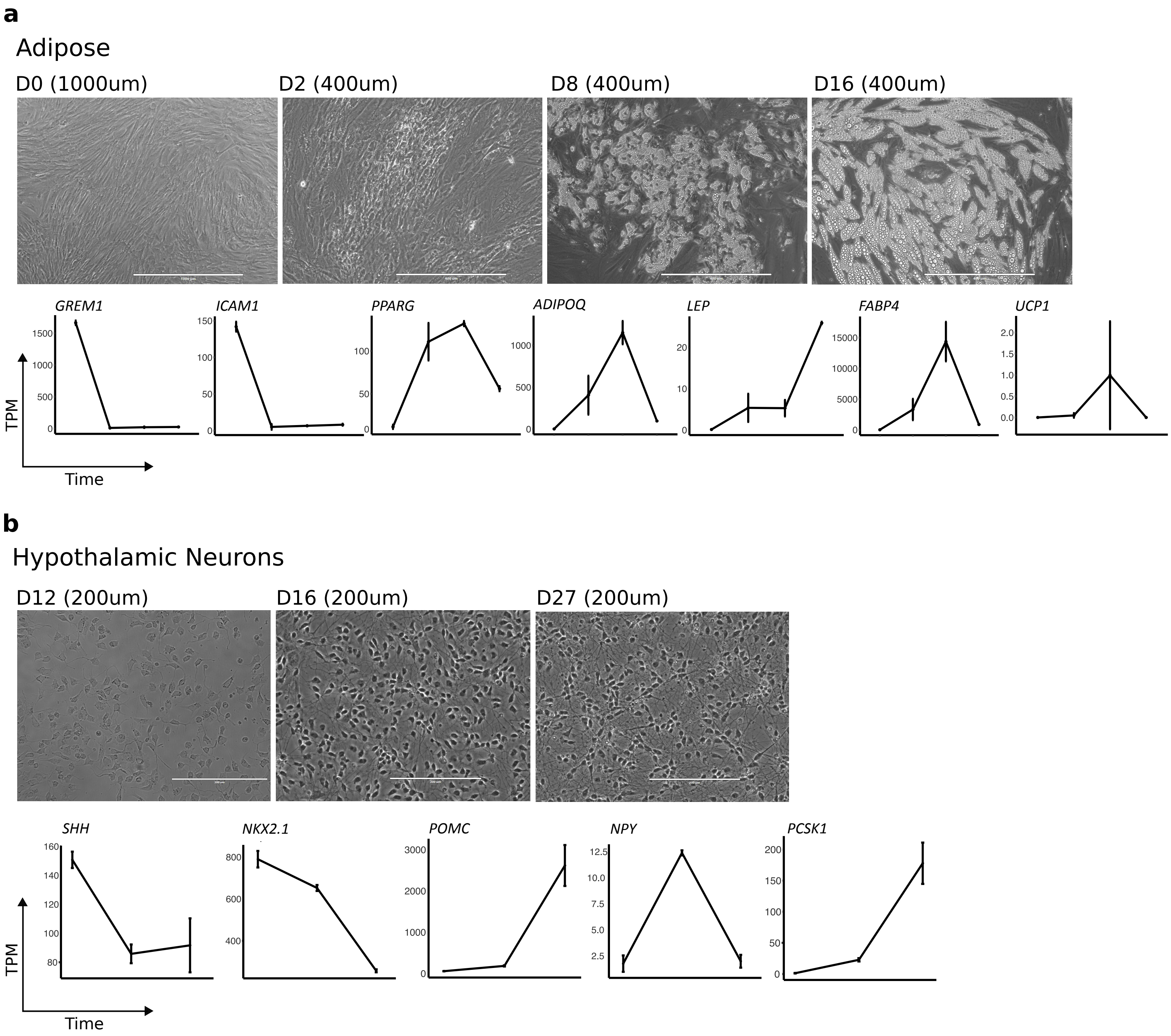

Supplementary Figure 2

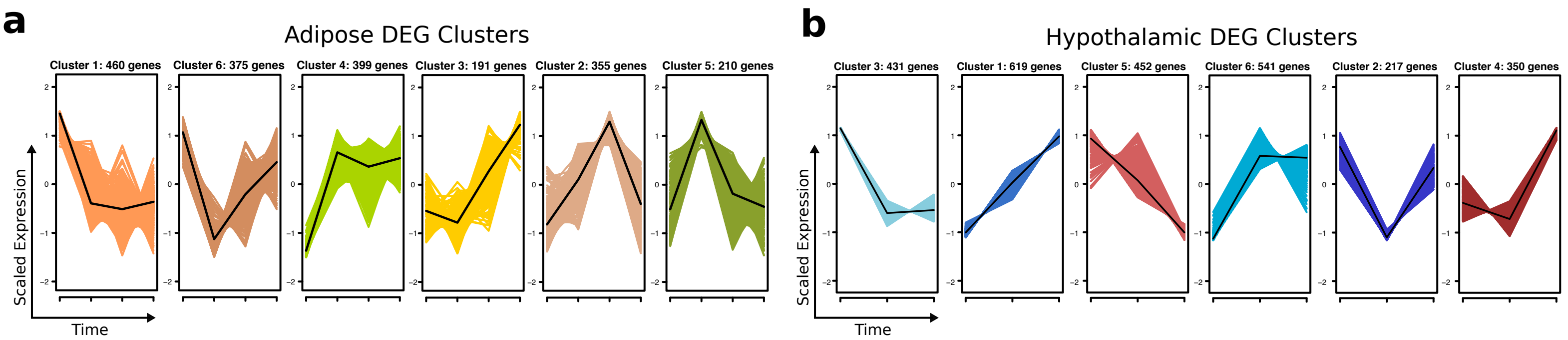

c

cHi-C	Preadipocytes D0	Differentiation Induction D2	Early Mature D8	Mature D16	Early Neuron D12	Neuron D16	Mature Neuron D27
Number of interactions	935,217	715,192	601,109	617,053	596,847	124,750	379,531
Median interaction length	197,962 bp	188,937 bp	178,058 bp	179,213 bp	180,139	188,383	235,048

ATAC-seq	Preadipocytes D0	Differentiation Induction D2	Early Mature D8	Mature D16	Early Neuron D12	Neuron D16	Mature Neuron D27
Number of peaks	144,540	79,955	69,745	24,023	122,311	46,083	72,821

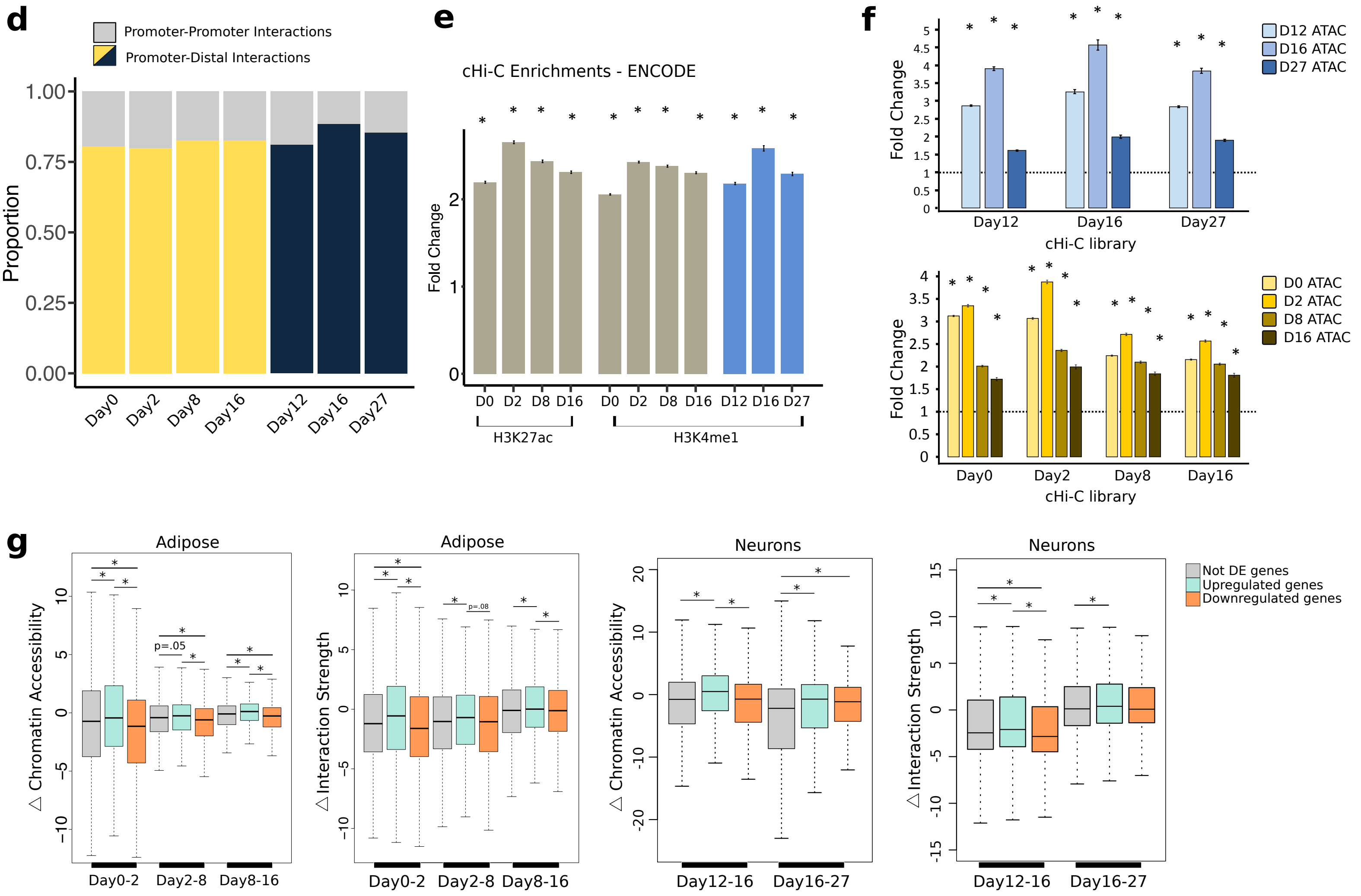

Supplementary Figure 3

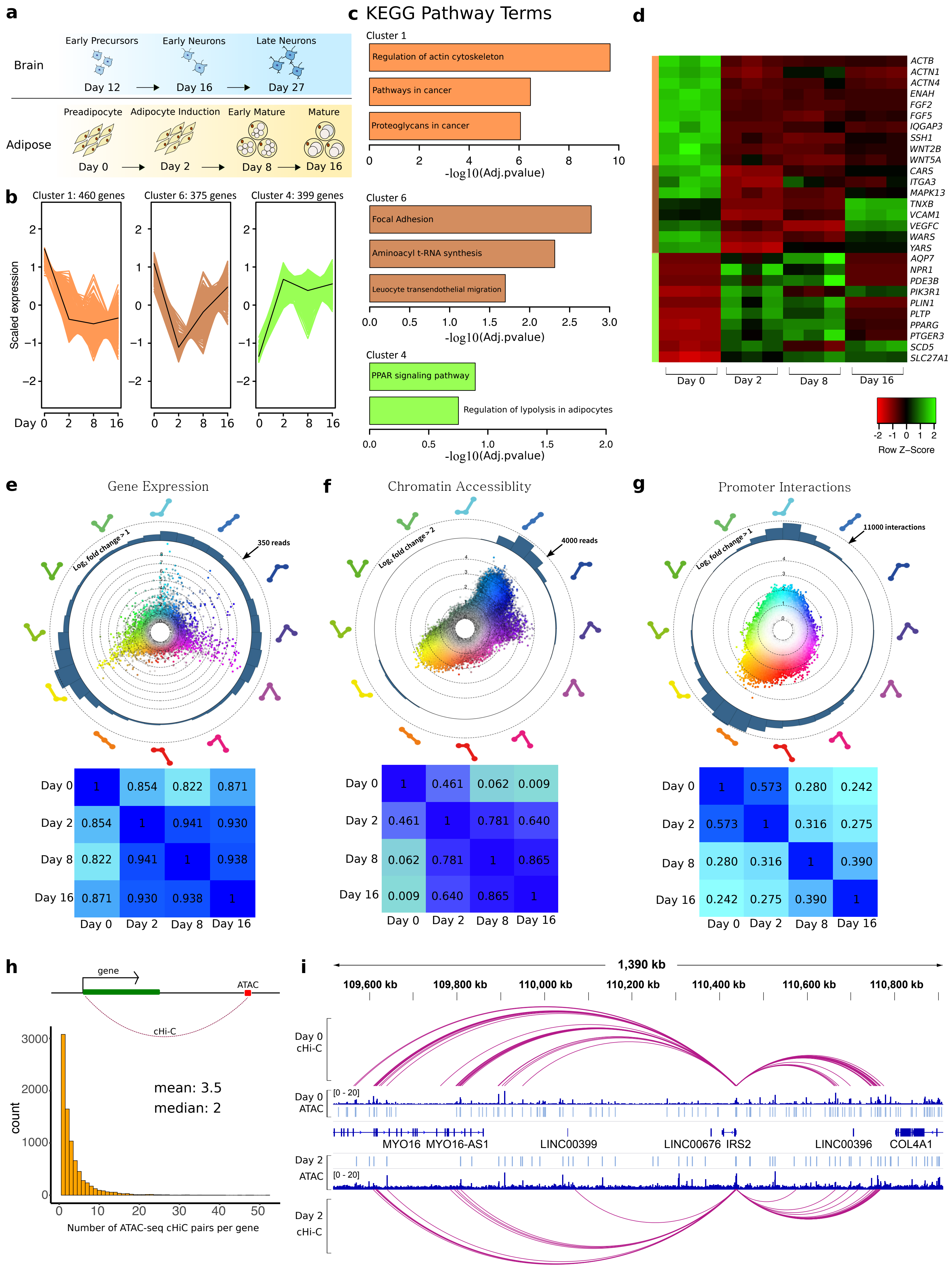

Supplementary Figure 4

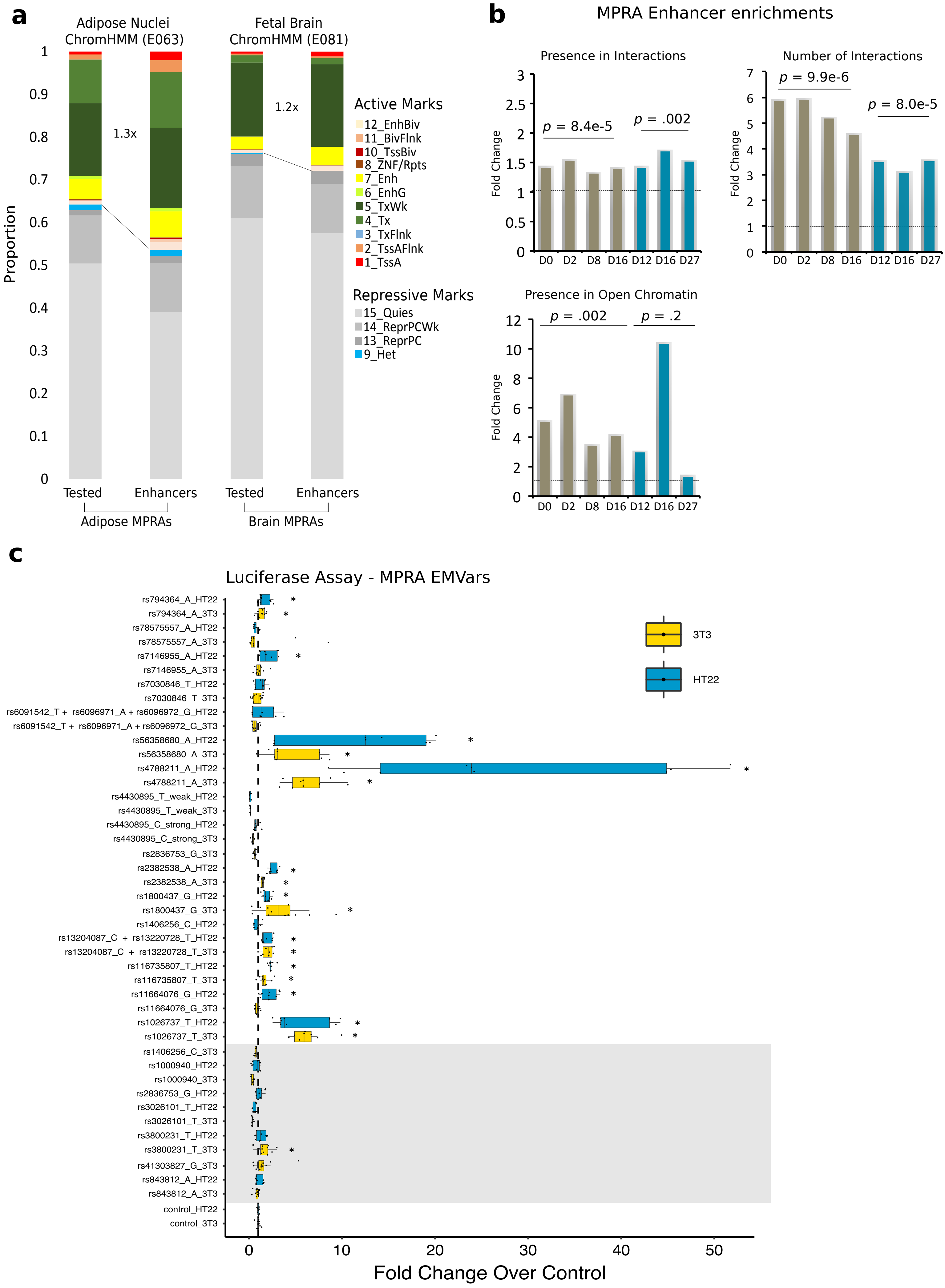

Supplementary Figure 5

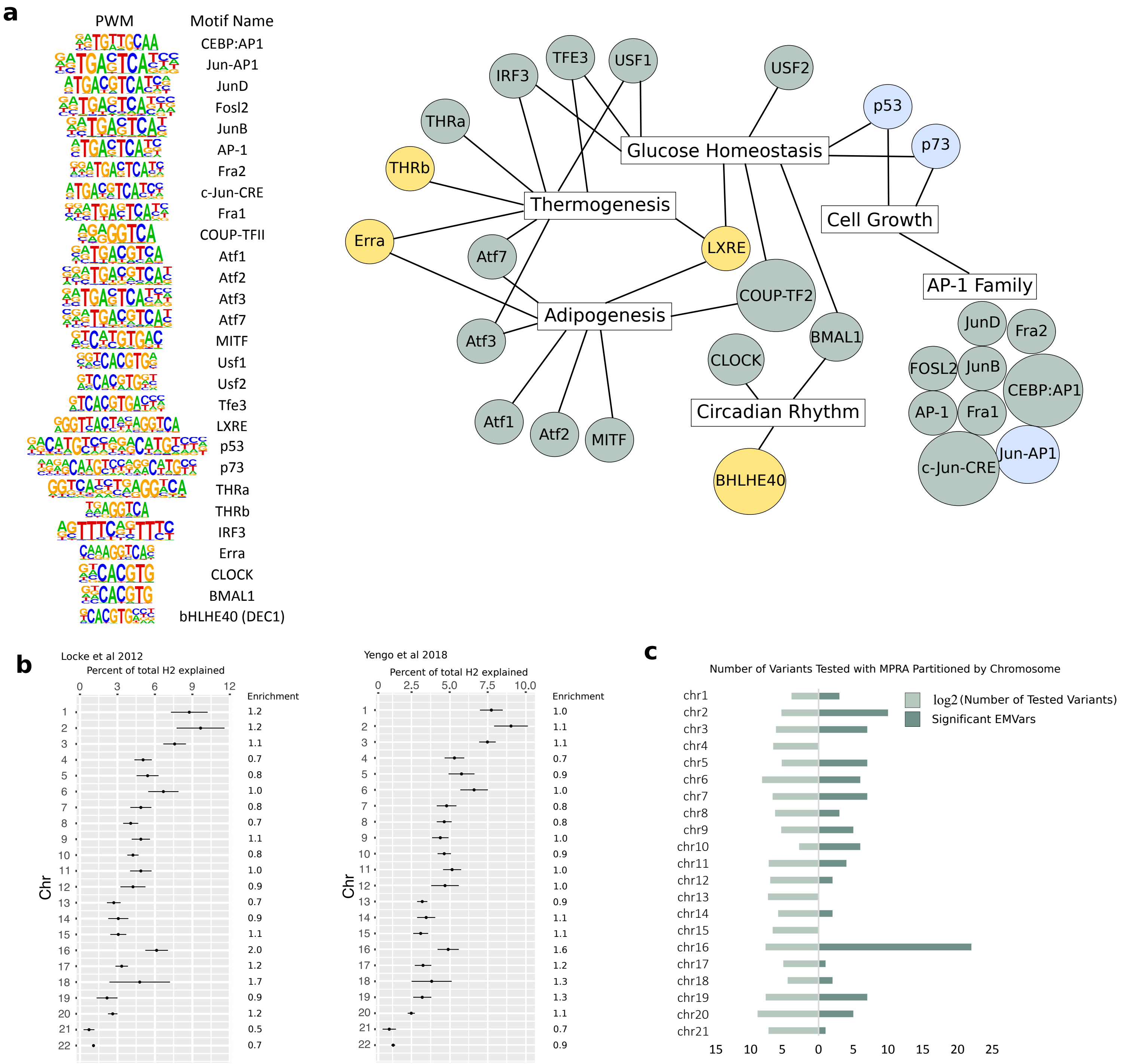

Supplementary Figure 6

a

Locus	SNP	GTEx eQTL	cHi-C interaction	Brain EMVar	Adipose EMVar
SBK1 locus	rs2650492	X	X	X	X
	rs28685654				X
	rs6498084			X	
ATP2A1 locus	rs56358680	X	X	X	X
	rs57719896	X	X	X	
	rs9972768	X	X	X	X
	rs4788100	X	X		X
	rs12446589	X	X	X	X
	rs7206214	X	X		X
	rs62037414	X	X	X	

RS Number	Position (GRCh37)	Allele Frequencies	Haplotypes			
rs56358680	chr16:28843118	A=0.636, G=0.364	A	G	G	A
rs55719896	chr16:28846866	G=0.636, A=0.364	G	A	A	G
rs9972768	chr16:28861734	A=0.636, C=0.364	A	C	C	A
rs4788100	chr16:28864673	T=0.636, C=0.364	T	C	C	T
rs12446589	chr16:28870962	G=0.636, A=0.364	G	A	A	G
rs3888190	chr16:28889486	C=0.636, A=0.364	C	A	A	C
rs7206214	chr16:28919341	G=0.667, A=0.333	G	A	G	A
rs62037414	chr16:28923521	T=0.672, C=0.328	T	C	T	C
		Haplotype Count	124	63	8	2
		Haplotype Frequency	0.6263	0.3182	0.0404	0.0101

b

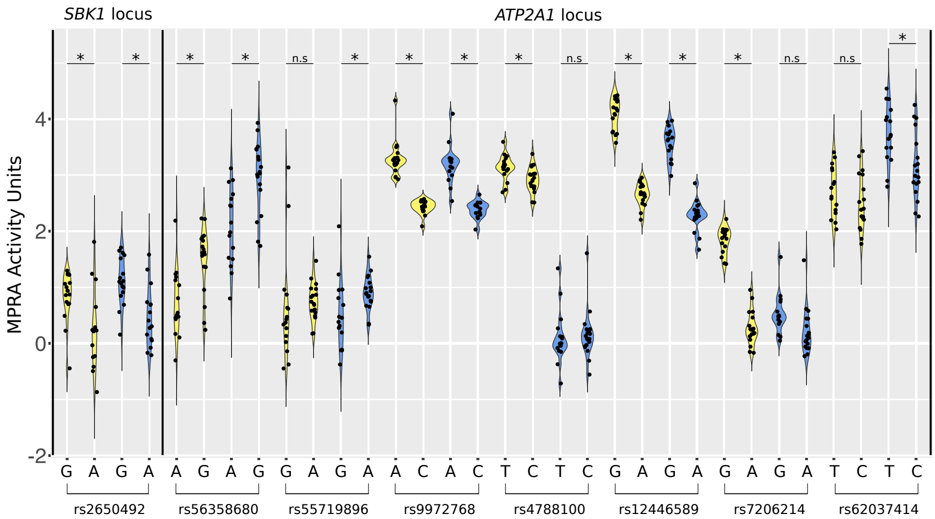

c

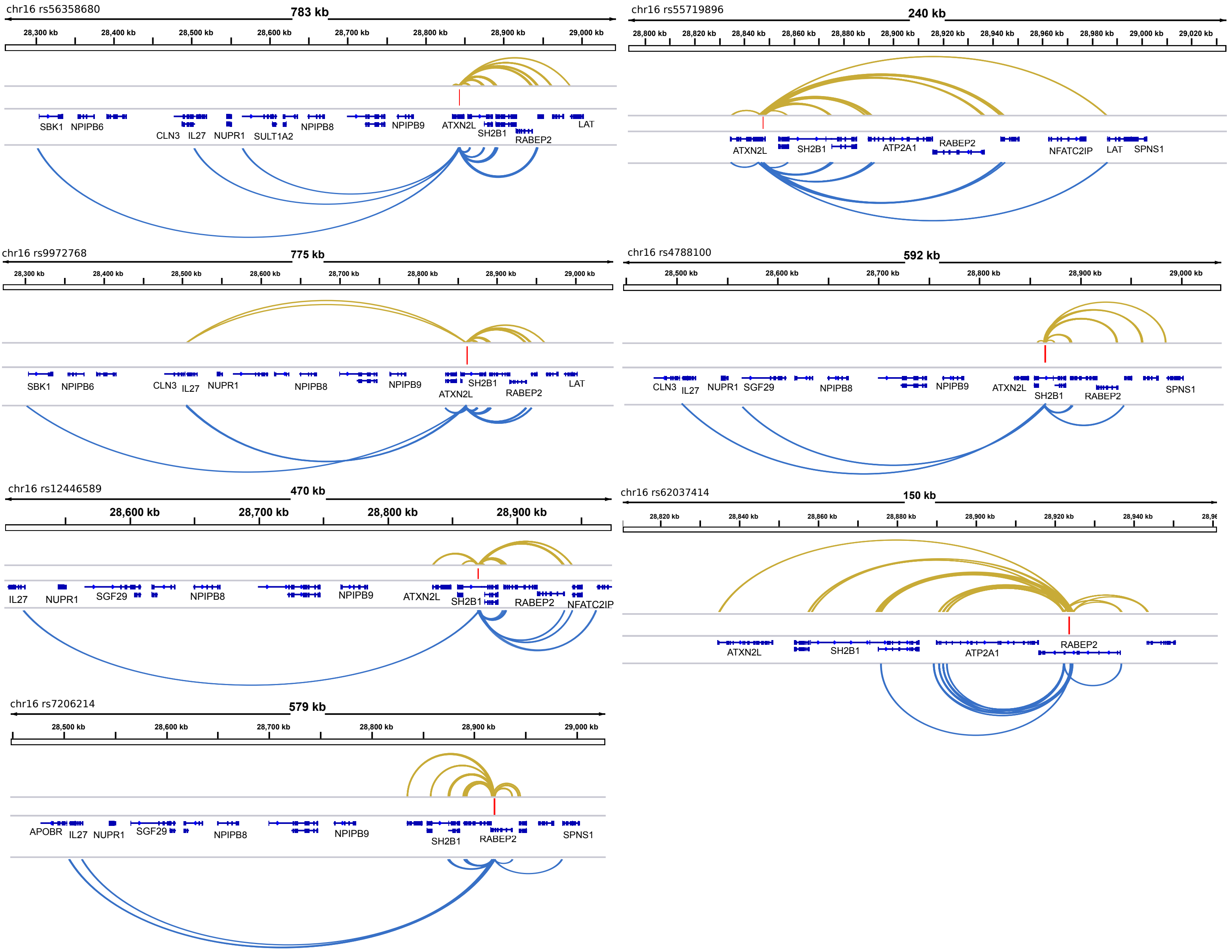

Supplementary Figure 7

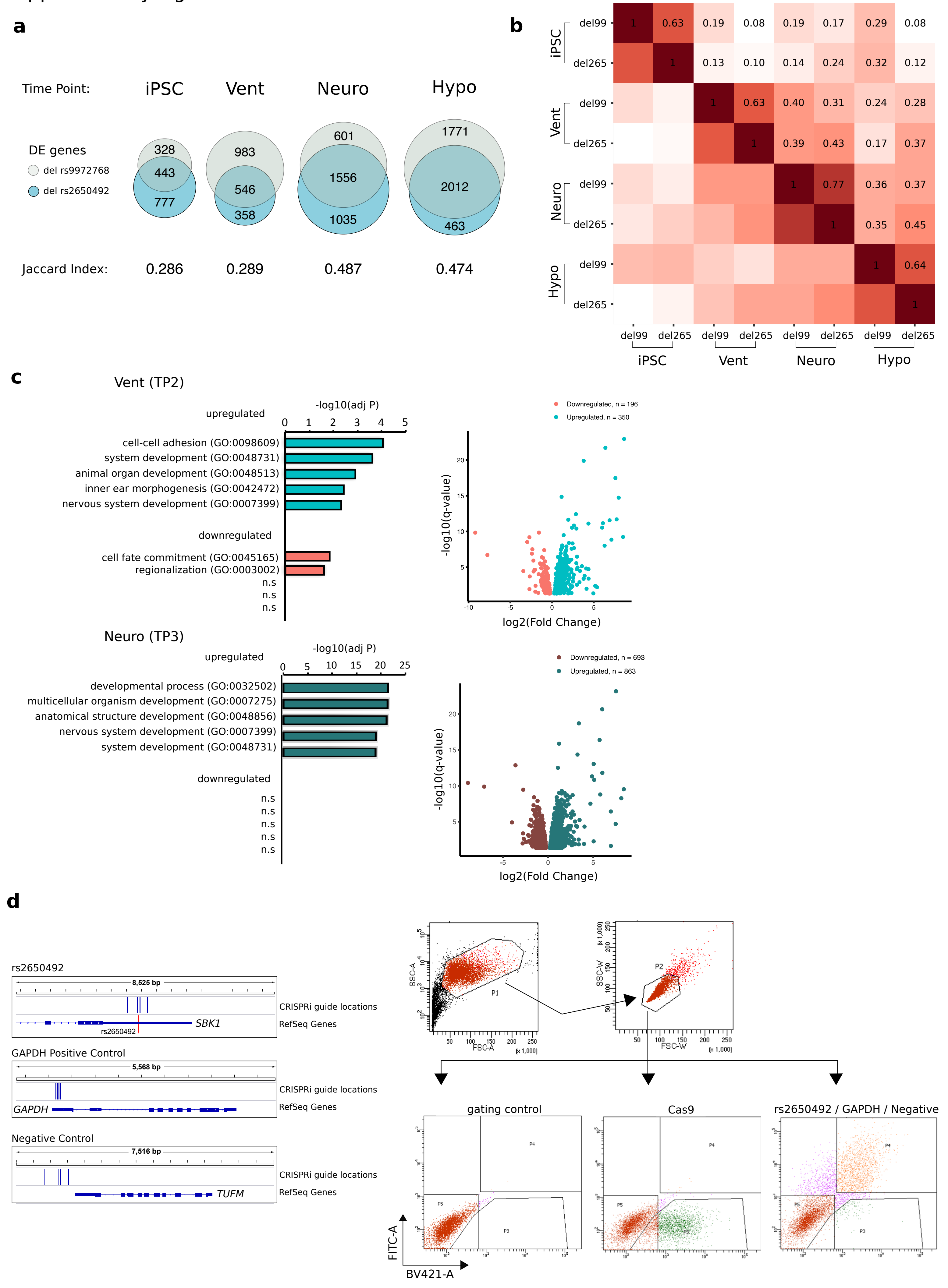

Supplement: Supplementary file 1 — Supplementary Information [file 41467_2021_25614_MOESM1_ESM.pdf]
