# Supplementary material for: A functional genomics pipeline identifies pleiotropy and cross-tissue effects within obesity-associated GWAS loci
Source: Nat Commun. 2021 Sep 6;12:5253. doi: 10.1038/s41467-021-25614-3 (PMC8421397; doi:10.1038/s41467-021-25614-3)
Supplement: Supplementary file 2 — Description of Additional Supplementary Files [file 41467_2021_25614_MOESM2_ESM.pdf]

Supplementary Data legends

Supplementary Data 1: Adipose Differentially Expressed Genes

This table contains all genes that were significantly differentially expressed during the adipocyte differentiation (FDR < 0.05) across any of our time points. *P* values were identified with limma and generated using a moderated *t*-test.

Supplementary Data 2: Hypothalamic Differentially Expressed Genes

This table contains all genes that were significantly differentially expressed during the hypothalamic differentiation (FDR < 0.05) across any of our time points. *P* values were identified with limma and generated using a moderated *t*-test.

Supplementary Data 3: All SNPs tested in the Massively Parallel Reporter Assay

This table contains all variants that were tested in the Massively Parallel Reporter Assay that were in high LD with the lead obesity GWAS variants identified in Locke et al 2015.

Supplementary Data 4: Significant MPRA Adipose Enhancers

This table contains all regions that were tested in the Massively Parallel Reporter Assay that were found to be significant enhancers in any adipose cell line (3T3-L1 preadipocytes, SGBS preadipocytes, SGBS mature adipocytes) $q < 0.05$ one-sided Mann-Whitney U test in at least half of all independent replicates.

Supplementary Data 5: Significant MPRA Brain Enhancers

This table contains all regions that were tested in the Massively Parallel Reporter Assay that were found to be significant enhancers in any brain cell line (HT22 or GT1-7) $q < 0.05$ one-sided Mann-Whitney U test in at least half of all independent replicates.

Supplementary Data 6: Significant EMVars

This table contains all significant enhancer modulating variants identified in any assayed cell type. *p* values were generated using a two sided Mann-Whitney U Test and adjusted for multiple comparisons using a 5% FDR cutoff

Supplementary Data 7: P-values from HOMER transcription factor enrichment analysis

This table contains all significantly enriched transcription factors identified by HOMER in MPRA enhancer regions. *p* values were generated by HOMER using a Binomial Test and were adjusted for multiple comparisons

Supplementary Data 8: Classes for genes within obesity GWAS loci stratified by cell type

This table contains the genes identified via this pipeline and are classified by cell type as well as level of support. The classes and their supporting data types are described in the methods.

Supplementary Data 9: CRISPRi guides

This table contains the guides used in the CRISPRi experiment

Supplementary Data 10: GO terms Enhancer Deletions

This table contains GO term information from the enhancer deletions related to nervous system development. Significance testing was performed using a Fishers Exact Test.

Supplementary Data 11: Primers and guides used in this study

This table contains primers and CRISPR guides used in this study.
