# Supplementary material for: A functional genomics pipeline identifies pleiotropy and cross-tissue effects within obesity-associated GWAS loci
Source: Nat Commun. 2021 Sep 6;12:5253. doi: 10.1038/s41467-021-25614-3 (PMC8421397; doi:10.1038/s41467-021-25614-3)
Supplement: Supplementary file 4 — Reporting Summary [file 41467_2021_25614_MOESM4_ESM.pdf]

nature research

Corresponding author(s): Amelia Joslin
Debora Sobreira
Marcelo Nobrega

Last updated by author(s): Jul 11, 2021

Reporting Summary

Nature Research wishes to improve the reproducibility of the work that we publish. This form provides structure for consistency and transparency in reporting. For further information on Nature Research policies, see our Editorial Policies and the Editorial Policy Checklist.

Statistics

For all statistical analyses, confirm that the following items are present in the figure legend, table legend, main text, or Methods section.

n/a	Confirmed	
☐	☒	The exact sample size (*n*) for each experimental group/condition, given as a discrete number and unit of measurement
☐	☒	A statement on whether measurements were taken from distinct samples or whether the same sample was measured repeatedly
☐	☒	The statistical test(s) used AND whether they are one- or two-sided<br>*Only common tests should be described solely by name; describe more complex techniques in the Methods section.*
☐	☒	A description of all covariates tested
☐	☒	A description of any assumptions or corrections, such as tests of normality and adjustment for multiple comparisons
☐	☒	A full description of the statistical parameters including central tendency (e.g. means) or other basic estimates (e.g. regression coefficient) AND variation (e.g. standard deviation) or associated estimates of uncertainty (e.g. confidence intervals)
☐	☒	For null hypothesis testing, the test statistic (e.g. *F*, *t*, *r*) with confidence intervals, effect sizes, degrees of freedom and *P* value noted<br>*Give P values as exact values whenever suitable.*
☒	☐	For Bayesian analysis, information on the choice of priors and Markov chain Monte Carlo settings
☒	☐	For hierarchical and complex designs, identification of the appropriate level for tests and full reporting of outcomes
☐	☒	Estimates of effect sizes (e.g. Cohen's *d*, Pearson's *r*), indicating how they were calculated

Our web collection on statistics for biologists contains articles on many of the points above.

Software and code

Policy information about availability of computer code

Data collection	BD FACSDiva 8.0.1
Data analysis	Promoter Capture Hi-C analysis<br>Promoter capture Hi-C reads were aligned to the genome using Bowtie2(v2.3.2) and technical artifacts were removed using HiCUP (v0.5.9). Significant interactions were detected over a background model of null expectation using CHiCAGO (v1.2.0). Only interactions with a CHiCAGO score > 5 at any time point were included in downstream analyses. Trans-chromosomal interactions and interactions between loci greater than 1 megabase apart were filtered from further analysis.<br><br>RNA-seq analysis<br>SGBS and hypothalamic neuron time course analysis: Gene-level read counts were quantified in each of the three technical replicates at each time point directly using salmon (v0.7.2), correcting for sequence-speficic bias and using a gene list derived from GENCODE release grch37.v19. Gene-level read counts were transformed into cpm and any gene with cpm < 1 in more than three samples across all time points was removed from further analysis. The data were normalized to account for library size using TMM normalization. Linear models testing pairwise differential expression between any two time points were then build using limma (v3.48.1) and tested using a moderated t-test accounting for mean-variance dependence and increased dispersion.<br><br>HEK293t CRISPRi analysis<br>Reads were mapped and gene counts were quantified with STAR (v2.5.1a). Counts were filtered to retain autosomal genes and exclude lowly expressed genes (< 1 CPM). PC1 clearly associated with sorting batch, so sorting batch was added as a covariate into the final linear model. P values were identified using glmQLFTest() from edgeR (3.28.1). Because of the very small effect sizes of CRISPRi in non-coding regions, cis-genes within the SBK1-ATP2A1 loci were considered for final significance testing. This list included all protein coding genes within these loci passing the expression threshold as well as GAPDH (14 genes total). Raw p values from these genes were Bonferroni corrected to get adjusted p values (P < 0.05/14 genes). Only genes passing the Bonferroni significance threshold were considered significantly affected by the CRISPRi

perturbations.

Enhancer deletion time course

Reads were mapped and gene counts were quantified with STAR (v2.5.1a). Counts were filtered to retain autosomal genes and exclude lowly expressed genes (< 1 cpm). Data clustered well by time-point and genotype so no batch effect correction was necessary. P values were identified using glmQLFTest() from edgeR. P values were FDR adjusted genome wide using p.adjust using the stats package (v3.6.2) in R.

ATAC-seq analysis

ATAC-seq reads were trimmed to remove Nextera adapters using cutadapt (v8.25) and aligned to the genome using Bowtie2 (v2.3.2). All reads mapping to the mitochondrial genome were removed from further analyses. Peak calling was performed using macs2 (v2.1.1.20160309) using no model and an extension size of 200. Significant peaks were considered those which survived FDR correction (q<0.05).

MPRA analysis

Barcode counts were extracted from fastq files and retained for downstream analysis only if they were exact matches with expected barcode sequences. Barcode count data was then analyzed for significance as previously described in Ulirsch et al. 2016. In brief, lowly expressed barcodes were removed and enhancer activity was determined from the remaining normalized counts using the following equation: Enhancer activity = log2 (output (CPM) - input (CPM)). Activity was then quantile normalized and enhancer p values were calculated using a one-sided Mann-Whitney U Test in R using the wilcox.test function. P values were corrected for multiple testing using the p.adjust function, method = “fdr”. All regions where at least one allele was determined to be a significant enhancer were then tested against the other allele of the variant for enhancer modulating effects using a two-sided Mann-Whitney U test in R with p values adjusted for multiple testing using the p.adjust function in R. Enhancer modulating variants were retained for downstream analyses if they were significant in half of all technical replicates or both biological MPRA replicates.

HSV plots

All Hue-Saturation-Value (HSV) analyses are developed from code originally published in Siersbaek et al21. Value (V) indicates the maximum log2(TPM/cpm) for a given gene at any time point, and so is defined as:

V=max(C_t)

Saturation (S) indicates the maximal fold change between any time points and is defined as:

S=1-(min_t (C_t))/V

Hue (H) indicates the pattern of change in gene expression across time, and is defined as:

H=60*(2+(C_0+C_2-C_16-V)/(V*S))*(C_2-C_0)/(|C_2-C_0 |)

For visualization purposes, the values of V and S were scaled between 0 and 1 based on rank.

For the purposes of HSV visualization, RNA-seq gene counts were converted to TPM to normalize for transcript length and then normalized by library size using trimmed mean of M-values (TMM) normalization and normalized by transcript length. Mean TPM was calculated at each time point, and all genes with mean log2(TPM) < 1 at any time point were removed from further analysis.

For ATAC-seq data the union set of significant peaks across time points was obtained, and peaks of a uniform length of 1kb were obtained by centering around the summit of the highest peak per peak locus in the union set. The counts per time point mapping to these 1kb union peaks were obtained and transformed to log2cpm format, normalizing by library size(defined as total number of reads in peaks per sample). Hue, saturation, and value were calculated using the same equations as with gene expression, using normalized log2(cpm) values as input. For promoter capture Hi-C data, counts were normalized by library size using (TMM) normalization and transformed into counts-per-million (cpm). Hue, saturation, and value were calculated using the same equations as with gene expression, using normalized cpm values as input.

Pearson’s r correlation analysis

Pearson correlations were performed on the normalized datasets used for HSV analysis with the cor function in R.

Fuzzy-c means clustering

Gene-level read counts were quantified in each technical replicate at each time point directly using salmon(v0.7.2), correcting for sequence-specific bias and using a gene list derived from GENCODE release grch37.v19. Gene-level read counts were transformed into cpm and any gene with cpm < 1 in more than three samples across all time points was removed from further analysis. The data were normalized to account for library size using TMM normalization. Linear models testing pairwise differential expression between any two time points were then build using limma and tested using a moderated t-test accounting for mean-variance dependence and increased dispersion in limma. All genes with significant differential expression between any two time points were included in the clustering analysis. Raw gene-level counts from salmon were normalized to account for transcript length and scaled to account for differences in gene expression across genes. Fuzzy c-means clustering was performed in R using the e1071 package. A gene was assigned to the cluster for which it had the highest membership if 1) its membership score was above 0.3 for the averaged replicates and 2) above 0.2 for each individual replicate. The top three clusters were defined by the highest average membership score.

LDSC Partitioned Heritability Analysis

Heritability per chromosome was calculated via LD score regression analysis using the ldsc command line tool (v1.0.0) using Locke et al 2012 BMI GWAS summary statistics and Yengo et al 2018 downloaded from the GIANT consortium. Briefly, .bim files from 1000 Genomes Phase 1 were downloaded and annotation files were created for each chromosome where chromosome was treated as a binary annotation. LD scores were then computed from these annotation files for input into partitioned heritability analysis. Summary statistics were filtered to contain only HapMap3 variants as advised.

Transcription factor motif analysis

All regions identified to be significant enhancers were included in this analysis. Regions were expanded to be 175bp (size of enhancers tested in MPRA) and then if two regions overlapped they were then merged so they would not become overrepresented in the analysis. The program findMotifsGenome.pl from HOMER (v4.8.3) was then used in addition to the -size flag to identify motifs that were overrepresented in significant MPRA enhancers from each cell line. These were compared to a size and base composition matched set of background sequences computed by HOMER to determine significance and p value. All p values from each cell line are included in the supplementary tables.

Calling MPRA EMVAR interactions with promoters
MPRA EMVars were considered to interact with a promoter if the distal end of the promoter interaction came within 1kb of the single base pair SNP location. EMVar SNP location and cHi-C BEDPE files were overlapped using the BEDtools (v2.27.1) pairToBed function.

Enrichment analysis:
The promoter-distal ends of interactions are enriched for functional histone mark (H3K27ac and H3K4me1) ChIP-seq peaks and ATAC-seq peaks compared to a distribution of randomly chosen, number-matched set of non-promoter MboI fragments within mappable genomic regions (N=100 iterations). The fold change of the observed overlap over our 100 randomized sets is presented. ChIP-seq datasets were obtained from Adipose Nuclei (E063) and Fetal Brain (E081) repositories from the Roadmap Epigenomics project.

IDT guide design tool (web tool): https://www.idtdna.com/site/order/designtool/index/CRISPR_SEQUENCE

MIT design tool (web tool): https://zlab.bio/guide-design-resources

CHOPCHOP (web tool): https://chopchop.cbu.uib.no

For manuscripts utilizing custom algorithms or software that are central to the research but not yet described in published literature, software must be made available to editors and reviewers. We strongly encourage code deposition in a community repository (e.g. GitHub). See the Nature Research guidelines for submitting code & software for further information.

Data

Policy information about availability of data

All manuscripts must include a data availability statement. This statement should provide the following information, where applicable:

- Accession codes, unique identifiers, or web links for publicly available datasets
- A list of figures that have associated raw data
- A description of any restrictions on data availability

All high throughput sequencing data that support the findings of this study have been deposited in https://www.ebi.ac.uk/arrayexpress/ with the following accession codes: rs9972768 and rs2650492 deletion RNA-seq (E-MTAB-10464) [https://www.ebi.ac.uk/arrayexpress/experiments/E-MTAB-10464/], MPRA (E-MTAB-10463) [https://www.ebi.ac.uk/arrayexpress/experiments/E-MTAB-10463/], ATAC-seq (E-MTAB-10462) [https://www.ebi.ac.uk/arrayexpress/experiments/E-MTAB-10462/], time course RNA-seq (E-MTAB-10461) [https://www.ebi.ac.uk/arrayexpress/experiments/E-MTAB-10461/], rs2650492 CRISPRi RNA-seq (E-MTAB-10460) [https://www.ebi.ac.uk/arrayexpress/experiments/E-MTAB-10460/] and in situ promoter capture Hi-C (E-MTAB-10488) [https://www.ebi.ac.uk/arrayexpress/experiments/E-MTAB-10488/].

Publically downloaded data used in this manuscript
ChIP-seq data can be downloaded from:
https://egg2.wustl.edu/roadmap/data/byFileType/peaks/consolidated/narrowPeak/E063-H3K4me1.narrowPeak.gz
https://egg2.wustl.edu/roadmap/data/byFileType/peaks/consolidated/narrowPeak/ E063-H3K27ac.narrowPeak.gz
https://egg2.wustl.edu/roadmap/data/byFileType/peaks/consolidated/narrowPeak/E081-H3K4me1.narrowPeak.gz
ChromHMM 15 state predictions can be downloaded from: https://egg2.wustl.edu/roadmap/data/byFileType/chromhmmSegmentations/ChmmModels/coreMarks/jointModel/final/E063_15_coreMarks_mnemonics.bed
https://egg2.wustl.edu/roadmap/data/byFileType/chromhmmSegmentations/ChmmModels/coreMarks/jointModel/final/E081_15_coreMarks_mnemonics.bed

Field-specific reporting

Please select the one below that is the best fit for your research. If you are not sure, read the appropriate sections before making your selection.

☒ Life sciences ☐ Behavioural & social sciences ☐ Ecological, evolutionary & environmental sciences

For a reference copy of the document with all sections, see nature.com/documents/nr-reporting-summary-flat.pdf

Life sciences study design

All studies must disclose on these points even when the disclosure is negative.

Sample size

No statistical methodology was used to determine the necessary sample size for the following experiments. However, we decided on replicate number based on previous publications which had success seeing statistical significance for measures of effect similar to what we wished to achieve with these experiments. These replicate numbers were based on the relative variance in the assay as well as the effect size we wished to achieve.

Promoter Capture Hi-C: Two technical replicates for each time point were performed derived from two unique differentiations for both SGBS and hypothalamic neurons. Each technical replicate was analyzed alone, and additionally the technical replicate raw sequencing data was merged and analyzed to produce merged datasets. Merged datasets and individual replicate datasets were used in downstream analyses.

ATAC-seq: Two technical replicates were performed for each time point derived from two unique differentiations for both SGBS and hypothalamic neurons. The two replicate sequencing data was merged and analyzed to produce merged datasets which were used in downstream analyses.

RNA-seq: Three technical replicates were performed derived from three unique differentiations for both SGBS and hypothalamic neurons.

MPRA: MPRA experiments were comprised of two soft biological replicates with 2-3 technical replicates per soft biological replicate. In total GT1-7 cells had 6 replicates, 3T3-L1 cells had 7 replicates, HT22 cells had 5 replicates, SGBS Day 0 cells had 6 replicates, and SGBS Day 8 cells had 5 replicates. A replicate was considered "biological" if the input DNA library was separately cloned from the beginning from our Agilent oligonucleotides.

Luciferase Assays: The luciferase assays used for MPRA validation and allele specific enhancer activities (supplementary figure 3 and Figure 4d) had between 3-12 replicates per construct, where different DNA preps were used and the cells were transfected, collected, and analyzed on different days. This varied based on technical variance within cell types, as different cell types had variable levels of transfection leading to higher variance and thus required more replicates to achieve sufficient power.

HEK293t CRISPRi: This experiment had 4-5 technical replicates per condition. GAPDH (5 replicates), rs2650492 (5 replicates), Negative Control (4 replicates). Replicates were transfections, FACS and collections that were performed on different days.

Enhancer deletion iPSC differentiation: This experiment had 3-4 homozygous biological replicate deletion clones of each genotype that were collected during four stages of differentiation from the iPSC stage to early neuronal precursor stage.

Data exclusions

No data were excluded from the analyses.

Replication

To generate reference omics maps we used three (RNA-seq) or two (ATAC-seq, HiC-seq) replicates of each assay at each time point representing two or three independent differentiations. Each replicate was performed successfully.
As described above, all omics experiments (RNA-seq, ATAC-seq, HiC-seq, and MPRA) were carried out and visualized with PCA to verify that cell types, differentiation stages, or perterbations (e.g genome editing) clustered as expected. Key cellular expression markers for each cell type were confirmed, and enhancer predictions were replicated with a variety of orthogonal techniques (histone mark enrichment, luciferase assay, ATAC-seq enrichments, etc) throughout the manuscript.

Enhancer deletion lines were confirmed for homozygous deletions via a combination of PCR genotyping and sanger sequencing. CRISPRi knockdown was confirmed using a positive control set of guides targeting the promoter of GAPDH. MPRA findings were validating using luciferase assays as described above.

Randomization

All experiments were performed with caution and randomized throughout to reduce potential for batch effects to emerge during analysis. For example, during the RNA-seq analysis, covariates such as RIN score, RNA-extraction batch, read mapping, FACS sorting date and RNA-library prep batch were correlated with all PCs that explained greater than 5% of the variance in the RNA-seq data using a linear model. If the covariate significantly correlated with one of these top PC's it was adjusted for when determining significantly differentially expressed genes. Covariates that were included in the model are outlined in the methods section.

Blinding

The experimental work in this manuscript was conducted by Amelia Joslin and/or Debora Sobreira. Data preprocessing and significance calling was conducted by Grace Hansen and/or Noboru Sakabe, who determined data quality and were made blind to experimental groups or perturbations as much as possible.

Reporting for specific materials, systems and methods

We require information from authors about some types of materials, experimental systems and methods used in many studies. Here, indicate whether each material, system or method listed is relevant to your study. If you are not sure if a list item applies to your research, read the appropriate section before selecting a response.

Materials & experimental systems

n/a	Involved in the study
☒	☐ Antibodies
☐	☒ Eukaryotic cell lines
☒	☐ Palaeontology and archaeology
☒	☐ Animals and other organisms
☒	☐ Human research participants
☒	☐ Clinical data
☒	☐ Dual use research of concern

Methods

n/a	Involved in the study
☒	☐ ChIP-seq
☐	☒ Flow cytometry
☒	☐ MRI-based neuroimaging

Eukaryotic cell lines

Policy information about cell lines

Cell line source(s)	HT22 – purchased from Millipore Sigma<br>GT1-7 - purchased from Millipore Sigma<br>3T3-L1 – purchased from ATCC<br>HEK293t – purchased from ATCC<br>SGBS – gift from Dr. Martin Wabitsch, these cells were originally derived from the stromal fraction of subcutaneous adipose from an individual with Simpson-Golabi-Behmel syndrome (SGBS). (Wabitsch et al. Int. J. Obes. Relat. Metab. Disord 2001)<br>iPSC – gift from Dr. Carole Ober and Dr. Yoav Gilad (Banovich et al. Genome Research 2018)
Authentication	cell lines were tested for cell-type specific markers using RNA-seq and/or qPCR
Mycoplasma contamination	all cell lines were tested regularly for mycoplasma contamination and were negative
Commonly misidentified lines<br>(See ICLAC register)	no commonly misidentified lines were used in this study

Flow Cytometry

Plots

Confirm that:

- [x] The axis labels state the marker and fluorochrome used (e.g. CD4-FITC).
- [x] The axis scales are clearly visible. Include numbers along axes only for bottom left plot of group (a 'group' is an analysis of identical markers).
- [x] All plots are contour plots with outliers or pseudocolor plots.
- [x] A numerical value for number of cells or percentage (with statistics) is provided.

Methodology

Sample preparation	Cells were transfected with plasmids expression BFP and/or GFP. After 48 hours cells were dissociated from tissue culture dish and resuspended into a FACS tube containing warm media. Cells were sorted directly from FACS tube into 1.5ml eppendorfs and spun down.
Instrument	The instrument used is a BD Biosciences FACS Aria Fusion 5-18
Software	BD FACSDiva 8.0.1 was used to sort positive cells, no analysis (i.e FlowJo) was performed
Cell population abundance	dCas9 condition BFP positives = 30-50% of parent population<br>BFP, GFP double positives = 20-40% of parent population
Gating strategy	Gate 1: FSC-A 50,000-250,000 x SSC-A 1,000-100,000<br>Gate 2: FSC-W 50,000-125,000 x FSC-W 50,000-125,000<br>Gate 3: BFP-BV421 1,000+ x GFP-FITC 1,000+ (double positives were collected for conditions and BFP single positives were collected for dCas9 only control)

- [x] Tick this box to confirm that a figure exemplifying the gating strategy is provided in the Supplementary Information.
